# Is symptom duration before DMARD therapy a determinant of direct and indirect costs in DMARD-naïve RA patients? A systematic review

**DOI:** 10.1093/rap/rkad040

**Published:** 2023-04-13

**Authors:** Ilfita Sahbudin, Ruchir Singh, Jeanette Trickey, Aliaksandra Baranskaya, Alexander Tracy, Karim Raza, Andrew Filer, Sue Jowett, Annelies Boonen

**Affiliations:** Rheumatology Research Group, Institute of Inflammation and Ageing, University of Birmingham, Birmingham, UK; NIHR Birmingham Biomedical Research Centre, University Hospitals Birmingham NHS Foundation Trust and University of Birmingham, Birmingham, UK; Rheumatology Research Group, Institute of Inflammation and Ageing, University of Birmingham, Birmingham, UK; NIHR Birmingham Biomedical Research Centre, University Hospitals Birmingham NHS Foundation Trust and University of Birmingham, Birmingham, UK; Department of Rheumatology, Sandwell and West Birmingham NHS Trust, Birmingham, UK; Rheumatology Research Group, Institute of Inflammation and Ageing, University of Birmingham, Birmingham, UK; NIHR Birmingham Biomedical Research Centre, University Hospitals Birmingham NHS Foundation Trust and University of Birmingham, Birmingham, UK; Rheumatology Research Group, Institute of Inflammation and Ageing, University of Birmingham, Birmingham, UK; NIHR Birmingham Biomedical Research Centre, University Hospitals Birmingham NHS Foundation Trust and University of Birmingham, Birmingham, UK; Rheumatology Research Group, Institute of Inflammation and Ageing, University of Birmingham, Birmingham, UK; NIHR Birmingham Biomedical Research Centre, University Hospitals Birmingham NHS Foundation Trust and University of Birmingham, Birmingham, UK; Rheumatology Research Group, Institute of Inflammation and Ageing, University of Birmingham, Birmingham, UK; NIHR Birmingham Biomedical Research Centre, University Hospitals Birmingham NHS Foundation Trust and University of Birmingham, Birmingham, UK; Department of Rheumatology, Sandwell and West Birmingham NHS Trust, Birmingham, UK; Rheumatology Research Group, Institute of Inflammation and Ageing, University of Birmingham, Birmingham, UK; NIHR Birmingham Biomedical Research Centre, University Hospitals Birmingham NHS Foundation Trust and University of Birmingham, Birmingham, UK; Health Economics Unit, Institute for Applied Health Research, University of Birmingham, Birmingham, UK; Division of Rheumatology, Department of Internal Medicine, Maastricht University Medical Center, Maastricht, The Netherlands; Care and Public Health Research Institute (CAPHRI), Maastricht University, Maastricht, The Netherlands

**Keywords:** RA, early diagnosis, direct/indirect costs, health economic outcomes

## Abstract

**Objective:**

Early treatment of RA improves clinical outcomes; however, the impact on health economic outcomes is unclear. This review sought to investigate the relationship between symptom/disease duration and resource utilization/costs and the responsiveness of costs following RA diagnosis.

**Methods:**

A systematic search was performed on Pubmed, EMBASE, CINAHL and Medline. Studies were eligible if patients were DMARD-naïve and fulfilled 1987 ACR or 2010 ACR/EULAR RA classification criteria. Studies had to report symptom/disease duration and resource utilization or direct/indirect costs as health economic outcomes. The relationships between symptom/disease duration and costs were explored.

**Results:**

Three hundred and fifty-seven records were identified in a systematic search; nine were eligible for analysis. The mean/median of symptom/disease duration in studies ranged between 25 days and 6 years. Annual direct costs of RA following diagnosis showed a U-shaped distribution in two studies. Longer symptom duration before starting a DMARD (>180 days) was associated with lower health-care utilization in the first year of RA diagnosis in one study. Annual direct and indirect costs 6 months before RA diagnosis were higher in patients with shorter symptom duration (<6 months) in one study. Given the clinical and methodological heterogeneities, the association between symptom/disease duration and costs after diagnosis was not computed.

**Conclusion:**

The association between symptom/disease duration at the time of DMARD initiation and resource utilization/cost in patients with RA remains unclear. Health economic modelling with clearly defined symptom duration, resource utilization and long-term productivity is vital to address this evidence gap.

Key messages

- The association between symptom/disease duration before DMARD initiation and health economic outcomes in RA is unclear.
- Clinical and methodological heterogeneities impede direct comparison of health economic outcomes across RA studies.
- Longitudinal studies with defined symptom duration and long-term RA-associated costs will address this research question.

## Introduction

The impact of early treatment on clinical outcomes in RA is well reported [1]. However, the impact of early treatment on health economic outcomes is less clear. Patients with RA treated with intensive DMARD were more likely to stay in the workforce long term [2, 3]. This might result long term in overall lower indirect costs (i.e. lower loss of productivity). However, diagnostic decisions are vulnerable to false-positive and false-negative results. The consequence of over-diagnosis and over-treatment might lead to overall higher direct costs (i.e. higher medical costs) in the longer run, which might offset the cost savings made from improved productivity. Therefore, long-term economic diagnostic and treatment decision models are required to inform the optimal threshold for diagnostic/treatment decisions from an economic perspective. This will facilitate the estimation of long-term RA-related costs.

Therefore, as a first step, the relationship between symptom/diagnosis duration at the time of DMARD initiation and subsequent resource utilization/costs needs to be identified. We sought to investigate this through a systematic review of cost-of-illness and cost-effectiveness studies of DMARD-naïve RA patients.

## Methods

The full Methods section is detailed in Supplementary Data S1, available at *Rheumatology Advances in Practice* online.

### Protocol and registration

The protocol was registered on the International Prospective Register of Systematic Reviews (PROSPERO 2017 CRD42017077593); https://www.crd.york.ac.uk/prospero/display_record.php?ID=CRD42017077593.

### Study identification/search strategy

PubMed, EMBASE, CINAHL and Medline electronic databases were searched up to 25 January 2023. All systematic searches were conducted using the same search terms and strategy (Supplementary Data S2, available at *Rheumatology Advances in Practice* online). Additional records were identified through independent manual database searching, external sources and reference scanning of relevant retrieved full-text articles. Study selection, data extraction and quality assessment were done independently by two authors (I.S. and R.S.); discrepancies were resolved by consensus or through a third reviewer (A.Bo.). Table 1 shows the PICOT framework.

**Table 1.** PICOT framework to capture studies cost or resource utilization as an outcome by symptom or disease duration in patients with DMARD-naïve RA

Population	DMARD-naïve RA

Intervention	Any DMARDs

Comparator	Any other DMARD treatment

Outcome	Direct costs Medication costs Indirect costs Productivity costs Resource use

Time	Duration immediately preceding study inclusion or DMARD start or the period following it

Context	Disease or symptom duration in relationship to the costs/resources

PICOT: patient, intervention, comparison, outcome and time.

### Study selection

Study inclusion criteria were as follows: aged ≥18 years and fulfilling the 1987 ACR or 2010 ACR/EULAR RA classification criteria; DMARD-naïve; symptom/disease duration reported; cross-sectional and longitudinal study; and health economic outcomes reported as costs or resource utilization. Studies excluded were studies of non-RA inflammatory arthritides and conference abstracts, systematic reviews and review articles.

### Data extraction

The following data were extracted: study characteristics; potential determinants of RA costs; sources of resource utilization and costs; and health economic outcomes.

### Quality assessment

The Strengthening The Reporting of Observational Studies in Epidemiology (STROBE) checklist [4] and a modified checklist by Drummond and Jefferson [5] were used for quality assessment.

### Data synthesis and statistical analysis

A meta-analysis/regression on the association between disease/symptom duration and costs could not be performed owing to the number of studies and methodological heterogeneity, especially in reporting of health economic outcomes. Cost data per patient per year for the reported duration in studies were recorded and summarized in a unifying currency of US Dollars 2021 after adjusting for the Purchasing Power Parity (PPP) and Consumer Price Index (CPI) 2021 [6, 7].

## Results

**Figure 1.**
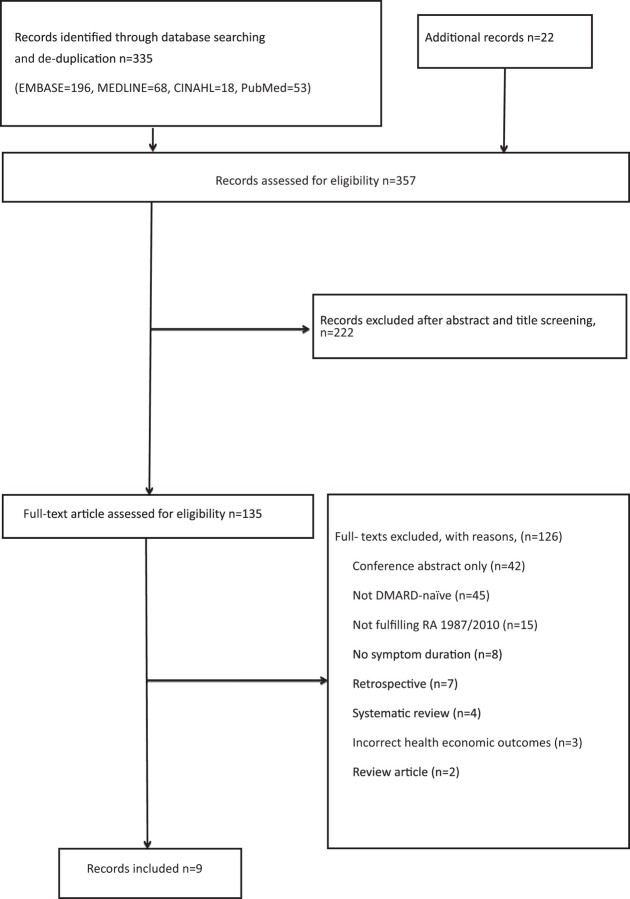
PRISMA flow chart of the four searches conducted. CINAHL: The Cumulative Index to Nursing and Allied Health; EMBASE: Excerpta Medica Database; MEDLINE: Medical Literature Analysis and Retrieval System Online

Nine articles were included in this systematic review. The Preferred Reporting Items for Systematic Reviews and Meta-Analyses (PRISMA) flow chart shows the literature search results (Fig. 1).

Table 2 summarizes study characteristics, cost categories and annual costs in international USD 2021. Six papers were cost-of-illness studies [8–13] and the remainder cost–utility studies [14–16]. Four studies were observational studies [8, 11, 13, 16] and five randomized controlled trials (RCTs) [9, 10, 12, 14, 15].

**Table 2.** Study characteristics, health economic outcomes and annual costs in US Dollars 2021

Author country, year	ObjectiveStudy designStudy setting	Patient characteristicsSymptom duration	OutcomeStudy perspective	Results as resources or costs by category (e.g. days hospitalized) or type (total health care; productivity)	Results as total resources or cost in local currency at time of the study	Cost per person per year in USD 2021 after adjusting for purchasing power parity and consumer price index 2021 (OECD, 2021) [1, 2]	
Luurssen-Masurel *et al.* [14] The Netherlands, 2021	Objective: to assess cost-effectiveness of three different initial treatments in seronegative DMARD-naïve RA patients, defined as patients from the tREACH trial with an intermediate probability of developing persistent arthritis who fulfilled RA 2010 criteria and were RF and ACPA negative at baseline Study design: cost–utility study in the context of clinical trial of 1 year duration. Study setting: patients recruited from eight rheumatology centres	*n*: 116 Female: 69.8% Age (average): 54.8 years Symptom duration, median (IQR): 134 (95–205) days	Outcomes: Incremental cost-effectiveness ratio between two of the three initial treatment strategies. Loss of productivity per year by: friction cost approach (including productivity loss owing to presenteeism) valued at age- and sex-dependent standard costs per hour. Study perspective: Partial societal Health care	Currency: Euros 2019 Total health-care costs by treatment strategy group per patient during 1 year of follow-up mean (s.d.) iMTX: 2584 (2196) iHCQ: 2123 (2172) iGC: 3050 (3461) Total productivity costs by treatment strategies group Mean (s.d.): iMTX: 8249 (14 171) iHCQ: 9085 (11 571) iGC: 7453 (10 446)	Total costs (health-care and productivity costs) by treatment strategy group per patient per year Mean: iMTX 10832 iHCQ 11 208 iGC 10 502	Total health-care costs by treatment strategy group, per patient in USD 2021 Mean: iMTX 3456 iHCQ 2839 iGC 4079 Total productivity costs by treatment strategies group in USD 2021 Mean: iMTX 11 031 iHCQ 12 149 iGC 9967 Total costs (health-care and productivity costs) by treatment strategy groups in USD 2021: Mean: iMTX 14485 iHCQ 14 988 iGC 14 ,044	

Verhoeven *et al.* [15] The Netherlands, 2021	Objective: to assess cost-effectiveness of initiating TCZ ± MTX *vs* initiating MTX as treat-to-target treatment strategies over 5 years in early DMARD-naïve RA. Study design: cost–utility study in the context of a clinical trial (2 years) and post-clinical trial follow-up (3 years). Study setting: 21 rheumatology outpatient clinics in the Netherlands	*n*: 317 Female, *n* (%): TCZ+MTX 65 (61) TCZ 78 (76) MTX 69 (64) Age, years, median (IQR): TCZ+MTX 53.0 (46.0–60.0) TCZ 55.0 (47.0–63.0) MTX 53.0 (44.5–62.0) Symptom duration, days, median (IQR): TCZ+MTX 24.5 (16.0–41.5) TCZ 25.5 (18.0–45.0) MTX 27.0 (15.0–46.0)	Outcomes: Incremental cost-effectiveness ratios between two treatment strategies. Productivity loss costs by human capital approach and friction cost approach. Study perspective: Health care Partial societal	Currency: Euros 2017 Costs (€, rounded to the nearest hundred) by treatment strategies group, means Medication costs: TCZ + MTX 17 900 TCZ 18 400 MTX 4400 Direct health-care costs (excluding medication costs): TCZ+MTX 6100 TCZ 7200 MTX 7000 Indirect non-health-care-related costs: TCZ+MTX 1100 TCZ 1600 MTX 1500 Productivity costs loss using human capital approach: TCZ+MTX 6700 TCZ 5600 MTX 6500 Productivity loss costs using friction cost approach: TCZ+MTX 2500 TCZ 2300 MTX 2500	Total costs (health-care and productivity costs) by treatment strategy group (in euros 2017) Mean per patient per year, at end of year 1 Direct healthcare-related costs: TCZ+MTX 6100 TCZ 7200 MTX 7000 Total medication costs: TCZ + MTX 17 900 TCZ 18 400 MTX 4400 Total productivity costs loss using human capital approach: TCZ+MTX 6700 TCZ 5600 MTX 6500 Total productivity loss costs using friction cost approach: TCZ+MTX 2500 TCZ 2300 MTX 2500 Indirect non-health-care-related costs: TCZ+MTX 1100 TCZ 1600 MTX 1500	Total costs (health-care and productivity costs) by treatment strategies group (in USD 2021) Mean per year, at end of year 1 Direct health-care costs (excluding medication costs): TCZ + MTX 15 546 TCZ 18 350 MTX 17 840 Total medication costs: TCZ + MTX 45 620 TCZ 46 894 MTX 11 214 Total productivity costs loss using human capital approach: TCZ + MTX 17 076 TCZ 14 272 MTX 16 566 Total productivity loss costs using friction cost approach: TCZ + MTX 6371 TCZ 5862 MTX 6371 Indirect non-health-care-related costs: TCZ + MTX 2803 TCZ 4078 MTX 3823	

Syngle *et al.* [16] India, 2017	Objective: to assess the cost and effects of synthetic DMARDs in treatment-naïve RA patients. Study design: cost–utility study in the context of longitudinal observational study. Study setting: one rheumatology outpatient clinic	*n*: 98 Female: 86% Age, mean (s.d.): 47.8 (12.3) years Disease duration at inclusion, mean (s.d.): 5.8 (5.0) years	Outcome: average cost-effectiveness ratio. Cost is measured in monetary value and the effectiveness of treatment is measured as change in HAQ-DI. Study perspective: healthcare	Currency: Indian Rupees 2017 Direct medical costs Medication costs (average/month): DMARDs 398 CSs 136.3 NSAIDs 16.66 Medicines to prevent adverse drug reaction 48.8 Monitoring costs (average/month): Laboratory costs 354 Radiology 24.3 Ophthalmology 5.97 Doctor consultation charges (average/month): 10	Average direct medical costs per RA prescription per month in Indian Rupees 2017: 997 Average direct medical cost per patient per year in Indian Rupees (2017): 11 965	Total health-care (drugs and monitoring) cost per patient per year adjusted to USD 2021: 1008	

Kuijper *et al.* [8] The Netherlands, 2014	Objective: comparison of disease burden between RA patients and arthralgia in an early arthritis cohort. Study design: inception cohort study. Study setting: patients recruited at first consultation with general practitioners or Rheumatology outpatient of five hospitals.	*n*: 244^c^ Female: 68% Age, mean (s.d.): 54 (13.7) years Symptom duration at study inclusion^d^, mean (IQR): 103 (7–373) days	Outcome: Health-care utilization (number of visits): GP Specialist Physiotherapist Alternative Study perspective: health care	Health-care utilization At baseline (number of visits): GP 2.8 visits Specialist 1.4 Physiotherapist visits/5 = 0.5 Alternative visits 0.1 All visits 4.7 At 6-month time point: GP 0.5 Specialist 2.6 Physiotherapist visits/5 = 0.6 Alternative 0.1 All visits 3.9 At 12-month time point: GP 0.4 Specialist 1.6 *Physiotherapist visits/5 = 0.5 Alternative 0.1 All visits 2.6	Total health-care utilization units for the first 12 months post DMARD initiation: 6.5 visits per patient per year	Monetary value not reported	

Puolakka *et al.* [9] Finland, 2009^e^	Objective: to assess the impact of HAQ on productivity loss in early RA patients. Study design: data collection at 5-year follow-up in an extension of a randomized controlled trial. Study setting: 18 recruitment centres for FIN-RACo Trial.	HAQ group 1 *n*: 13 Female: 31% Age, mean (s.d.): 45 (9) years Disease duration at inclusion, mean (s.d.): 11 (9) months	Outcome: Work disability days Indirect costs^g^; Loss of productivity per year by: Human capital approach Friction cost approach Study perspective: partial societal	Values are given as mean per patient per year (95% CI) HAQ group 1 Work disability (days per year): 34 (5–145) Loss of productivity per year (HCA), euros: 440 (137–896) Loss of productivity per year (FCA), euros: 353 (118–712)		Loss of productivity costs per patient per year in USD 2021, mean: HCA 736 FCA 590	
		HAQ group 2 *n*: 65 Female: 62% Age, mean (s.d.): 45 (9) years Disease duration at inclusion, mean (s.d.): 8 (5) months		HAQ group 2 Work disability (days per year): 33 (19–57) Loss of productivity per year (HCA), euros: 2704 (1457–4606) Loss of productivity per year (FCA), euros: 1360 (963–1870)		Loss of productivity costs per patient per year in USD 2021, mean: HCA 4523 FCA 2275	
		HAQ group 3 *n*: 65 Female: 68% Age, mean (s.d.): 47 (4) years Disease duration at inclusion, mean (s.d.): 8 (5) months		HAQ group 3: Work disability (days per year): 146 (112–185) Loss of productivity per year (HCA), euros: 12 072 (8788–15 758) Loss of productivity per year (FCA), euros: 2452 (1902–3153)		Loss of productivity costs per year in USD 2021, mean: HCA 20 191 FCA 4101	
		HAQ group 4 *n*: 16 Female: 69% Age: 50 (s.d. 9) Disease duration at inclusion, mean (s.d.): 10 (7) months		HAQ group 4: Work disability (days per year): 272 (194–328) Loss of productivity per year (HCA), euros: 23 985 (16 448–33 141) Loss of productivity per year (FCA), euros: 3662 (2518–5237)		Loss of productivity costs per year in USD 2021, mean: HCA 40 116 FCA 6125	
Verstappen *et al.* [10] The Netherlands, 2004	Objective: to estimate annual direct costs and their predictors in patients with four disease duration groups. Study design: cost-of-illness study within open-label extension of two randomized clinical trials. Patients in RCT 1 were randomly assigned to one of four treatment regimes^b^. Patients in RCT 2 were allocated to either intensive or conservative MTX treatment. (Questionnaires were sent out in October 1999 and April 2000.) Study setting: seven rheumatology outpatient clinics in the Utrecht region^a^	*n*: 509 *n*: 96 from group with disease duration follow-up: 0 to ≤2 years Female: 73% Age, mean (s.d.): 54 (15) years Disease duration at inclusion, mean (s.d.): 0.9 (0.6) years	Outcome: Direct medical costs Consultations with health-care workers Admissions to health-care facilities (hospital, including surgical procedures, rehabilitation centre, nursing home) Medication Laboratory tests Devices to perform daily activities and adaptations at home Alternative medicine Other costs Study perspective: Health care and patient	Currency: Euros; publication year 2004. Mean (median) (range): Consultation with healthcare workers 1448 (1433) (0–8090) Admission to care facilities 1391 (7283) (0–57 930) RA-related medication 478 (406) (0–2895) Devices and adaptations 963 (2247) (0–15 571) Laboratory tests 296 (131) (75–975) Alternative therapies 103 (338) (0–6080) Total extra costs 554 (1094) (0–6080)	Direct costs per patient per year Mean (median) (range): 5235 (2923) (570–74 080)		Mean of total direct costs per patient per year in USD 2021: 14 613 Median of total direct costs per patient per year in USD 2021: 8159

Merkesdal *et al.* [11] Germany, 2001	Objective: to assess the extent of indirect costs, changes in cost components, and correlations between changes in cost and social, clinical and occupational variables within the first 3 years of RA. Study design: longitudinal prospective observational study. Study setting: four rheumatology centres	*n*: 133 Female: 63 Age, mean (s.e.m.): 47 (0.8) years Disease duration at inclusion, mean (s.e.m.): 7 (0.3) months	Outcome: indirect costs Loss of productivity owing to: sick leave work disability other work loss Study perspective: partial societal	Currency: US dollars for the period 1994–1996 Mean (s.e.m.): Sick leave Time 0–time 2 10 530 (990) Time 2–time 3 2520 (580) Time 0–time 3 7640 (740) Work disability Time 0–time 2 1210 (360) Time 2–time 3 4570 (960) Time 0–time 3 2520 (550) Other work loss Time 0–time 2 840 (370). Time 2–time 3 2800 (780). Time 0–time 3 1590 (480). Definition of time points: Time 0 = joint swelling onset Time 2 = 12 months from study enrolment Time 3 = 24 months from study enrolment	Currency: US dollars for the period 1994–1996 Total productivity costs (sick leave, work disability and other work loss) Mean (s.e.m.): Time 0–time 2 12 580 (1030) Time 2–time 3 9890 (1210) Time 0–time 3 11 750 (1120)	Cost per person per year in USD 2021 after adjustment for purchasing power parity and Consumer Price Index 2021 Total productivity costs (sick leave, work disability and other work loss) Mean: Time 0–time 2 20 180 Time 2–time 3 15 865 Time 0–time 3 18 848	

Newhall-Perry *et al.* [13] USA, 2000	Objective: to examine direct and indirect costs of RA during the first year of disease. Study design: longitudinal observational study. Study setting: patients recruited at 26 rheumatology centres in western USA and Mexico (3 practices are University medical centres and 23 community practices).	*n*: 150 Female: 80% Age, mean (s.d.): 51 (13) years Disease duration at inclusion, mean (s.d.): 5.9 (2.9) months	Outcome: Direct costs Indirect costs Study perspective: Health care (direct costs) Partial societal (indirect costs)	Currency: US dollars 1994 Disease duration <6 months (*n* = 87) Mean (s.d.): Direct costs per month 240 (285) Medication costs: 62 (101) Health-care visits: 65 (69) Radiographs 65 (196) Laboratory tests: 27 (26) Hospitalizations: 00 Assistive devices: 3 (6) Non-traditional treatments 1 (3) In-home assistance 9 (47) Outpatient procedures 8 (49) Indirect costs per month 348 (567) Disease duration ≥6 months Mean (s.d.) Direct costs per month 144 (149) Medication costs: 43 (36) Health-care visits 37 (28) Radiographs 26 (30) Laboratory tests 13 (12) Hospitalizations 16 (97) Assistive devices 3 (11) Non-traditional treatments 2 (9) In-home assistance 3 (16) Outpatient procedures 1 (5) Indirect costs per month 188 (506)	Results in local currency and year of assessment Mean (s.d.):	Cost per person per year in USD 2021 after adjusting for purchasing power parity and Consumer Price Index 2021 Total costs (direct and indirect costs) of RA per year per patient for overall cohort, mean: 10 372 Direct costs per year per patient for overall cohort, mean: 4322 Indirect costs of per year per patient for overall cohort, mean: 6072 Cost by disease duration groups: Indirect costs <6 months, mean: 7520 Indirect costs ≥6 months, mean: 4063 Direct costs <6 months, mean: 5186 Direct costs ≥6 months, mean: 3112 Total RA costs (direct and indirect) <6 months, mean: 12 663 Total RA costs (direct and indirect) ≥6 months, mean: 7174	
					Total RA costs (direct and indirect cost/month) in patients with disease duration <6 months 586 (686) Total RA costs (direct and indirect cost/month) in patients with disease duration ≥6 months 332 (585)		

van Jaarsveld *et al.* [12] The Netherlands, 1998	Objective: estimation of: Annual direct RA related costs in the first 6 years. Sociodemographic and clinical predictors of these costs. Study design: cross-sectional data collection of direct costs for all patients recruited in RCT. [First patient in trial was enrolled 1990. Results were represented as the total group independent of the treatment arm. Study questionnaire sent in April 1996] Study setting: Six rheumatology centres in Utrecht region.	*n*: 363 *n*: 63 from patient with symptom duration at 1 year follow-up Female: 64% Age, median (range): 57 (19–84) years Disease duration at inclusion: 0–1 years	Outcome: Direct medical cost:Health-care workers costDays in care facilitiesMedicationMedication side effects monitoringAlternative medicine Direct non-medical costs:Devices and adaptations at homeOther costs: travel expenses, medication not provided by national health service, additional costs of energy, telephone and clothing, payments to friends for care, payment for help around the house, and other costs specified by the patients.Study perspective: health care and patient	Currency: Dutch florins; September 1997. Direct medical costs for disease duration 0–1 year Mean (s.d.) median per patient per year: Total direct cost 14 455 (20 411) 7370 Subtotal direct medical cost^f^ 9882 (1898) 4444 Consultations with health-care worker 3355 (3112) 2340 Days in care facilities 4620 (15 521) 0 Medication 1340 (682) 1170 Monitoring for side effects 484 (311) 416 Alternative medicine 83 (299) 0 Subtotal direct non-medical cost 4573 (8934) 2268 Adaptations and devices 2814 (6797) 150 Other costs 1759 (3101) 600	Direct medical cost for disease duration 0–1 year Mean (s.d.) median per patient per year in Dutch florins: Total direct costs 14 455 (20 411) 7370 Subtotal direct medical cost^f^ 9882 (1898) 4444 Subtotal direct non-medical cost 4573 (8934) 2268	Cost per person per year in USD 2021 after adjusting for purchasing power parity and Consumer Price Index 2021 Mean (median) per patient per year in USD 2021 (at the end of year 1 of follow-up): Total direct costs 24 094 (12 285) Subtotal direct medical cost^f^ 16 472 (7407) Subtotal direct non-medical cost 7623 (3780)	

a Collaborating in the Utrecht RA cohort study group.

b Pyramid, i.m. gold, MTX or HCQ.

c *n* = 330 arthralgia patients recruited.

d Median (range).

e Outcome data were split into four groups based on HAQ: group 1 (HAQ = 0 at baseline and 6 months); group 2 (HAQ > 0 at baseline, 0 at 6 months); group 3 (HAQ ≥ 0 at baseline, >0 but <1.0 at 6 months); and group 4 (HAQ ≥ 0 at baseline, ≥1.0 at 6 months).

f Subtotal of medical cost includes costs owing to contacts with health-care workers, days spent in care facilities, medication, monitoring for side effects and alternative medicine. Subtotal of non-medical direct cost includes costs of adaptations in the home, devices and other costs.

g HCA= mean productivity per day over a 5-year follow-up was calculated for each patient and multiplied by the cumulative number of their days off work to yield the patients' loss of productivity by the HCA. FCA= estimation of loss of productivity, with the assumption that someone replaces the disabled worker after the friction period, that the initial production level is restored, and that production losses are confined to the friction period. RA-related work disability days were obtained from the official register, divided by the duration (in years) of follow-up during which the patient had not retired owing to other diseases or because of age. All final cost column states the cost per person per year in USD 2021 after adjusting for purchasing power parity and Consumer Price Index 2021.

GP: general practitioner; IQR: interquartile range; RCT: randomized controlled trial; TCZ: tocilizumab; USD: US dollars.

Sociodemographic and clinical characteristics of patients are summarized in Supplementary Table S1, available at *Rheumatology Advances in Practice* online. Cost categories, source of cost reference and results in local currency are summarized in Supplementary Table S2, available at *Rheumatology Advances in Practice* online.

The symptom, disease or diagnosis duration variables reported at baseline varied. Two studies reported symptom duration [8, 14], six studies disease duration [9–13, 16] and one diagnosis duration [15]. Only one study clearly defined symptom duration: ‘first onset of joint swelling’ [11]. The remaining studies did not state the definitions of symptom, disease or diagnosis duration [8–15].

Resource utilization and cost data across studies were heterogeneous (Table 1). Three studies reported costs (i.e. monetary value) but not resource utilization [13, 15, 16]. One study reported resource utilization without monetary values [8]. Three studies reported resource utilization and costs [10, 12, 14]. Two studies reported costs data as loss of productivity costs [9, 11].

Direct medical costs were reported in six studies (two observational studies [13, 16] and four clinical trials [10, 12, 14, 15]). Two studies reported direct non-medical costs [10, 12]. Health-care utilization with no monetary value was reported in one study [8].

Loss of productivity (indirect cost) was recorded in four studies [9, 11, 14, 15]. Two studies calculated productivity loss using the human capital and friction cost approach [9, 15]. One study used only the human capital approach [11], and one study used only the friction cost approach [14].

Study perspective refers to the point of view adopted in the economic evaluations [17], i.e. who pays for the cost. Common study perspectives are the patient, health-care system or society. Three studies reported societal perspectives (i.e. health-care and productivity loss costs) [13–15]. Two studies reported a partial societal perspective (productivity loss costs) [9, 11], and two studies reported costs from the health-care perspective [8, 16]. In addition, two studies reported both health-care (direct medical costs) and patient perspectives [10, 12].

Quality assessment has been included in Supplementary Data S3 and Table S3, available at *Rheumatology Advances in Practice* online.

### Narrative synthesis

Luurssen-Masurel *et al.* [14] performed a cost–utility study in seronegative RA patients in the Rotterdam Early Arthritis Cohort (tREACH) trial. The median symptom duration was 134 days [interquartile range (IQR) 95–205 days]; follow-up duration was 1 year. Initial treatment strategies were MTX (iMTX) 25 mg once weekly, HCQ (iHCQ) 400 mg daily or a tapering course of oral glucocorticoids (iGC). There was no significant difference in the mean cumulative health-care costs over 1 year for treatment with iMTX, iHCQ and iGCs (Table 2). The difference in productivity costs over 1 year between the three groups was mainly attributed to different levels of presenteeism (Table 1). After adjusting for PPP and CPI 2021, mean total costs (health-care and productivity costs) by treatment strategy groups in USD 2021 were $14 485, $14 988 and $14 044 for the iMTX, iHCQ and iGC groups, respectively. The association between symptom duration and health-care/productivity costs in the overall cohort or by treatment groups was not assessed.

Verhoeven *et al.* [15] reported a 5-year cost–utility analysis of an RCT comparing tocilizumab (TCZ) plus MTX or TCZ monotherapy with MTX monotherapy in DMARD-naïve early RA patients. The median (IQR) symptom duration by treatment groups was 25 (16–42) days, 26 (18–45) days and 27 (15–46) days for the TCZ plus MTX, TCZ and MTX groups, respectively. Cumulative 5-year productivity cost loss [by human capital approach (HCA)] was highest in the TCZ plus MTX group (€51 700; *n* = 106) compared with the TCZ monotherapy and MTX monotherapy groups [€39 900; *n* = 103 and €46 500, *n* = 108 respectively]. Cumulative 5-year productivity cost loss (HCA) was highest in the TCZ plus MTX group (€51 700) compared with the TCZ monotherapy and MTX monotherapy groups (€39 900 and €46 500, respectively). After adjusting for PPP and CPI 2021, total direct health-care-related costs (mean) in USD 2021 at the end of year 1 were $15 546, $8350 and $17 840 per patient for the TCZ plus MTX, TCZ and MTX groups, respectively. The association between symptom duration and health-care or productivity costs in the overall cohort or by treatment groups was not assessed.

Syngle *et al.* [16] reported RA-related health-care costs in a single-centre prospective observational study of 3 months in India. The study assessed the cost-effectiveness of synthetic DMARDs in DMARD-naïve RA patients [16]. The mean disease duration was 5.78 years (s.d. 4.84 years). Costs reported were the average total direct medical cost per prescription per month over the 3-month study period. This figure equates to 997.05 Indian Rupees per patient. After adjusting for PPP and CPI 2021, the average (extrapolated) annual direct medical costs at the end of year 1 in USD 2021 was $1008 per patient. The association between disease duration and direct medical costs was not assessed.

Kuijper *et al.* [8] compared health-care utilization between arthralgia and DMARD-naïve early RA patients at baseline, 6 and 12 months in a Dutch inception observational cohort study [8]. The median symptom duration for RA patients was 103 days (range 7–373 days). Use of DMARDs was not reported. A longer (>180 days) *vs* short symptom duration (90–180 days) at baseline was associated with lower levels of health-care utilization over 12 months [Incidence Ratio Rate of 0.65 (95% CI 0.50, 0.85, *P* = 0.002)]. The mean number of visits to medical specialists peaked at 6 months in the RA group (Table 2). However, a decrease in overall health-care visits (i.e. general practitioner, medical specialist, physiotherapist and alternative health practitioner visits) was observed following diagnosis (Table 2). No monetary value was reported in this study. In summary, longer symptom duration (>180 days) was associated with lower health-care utilization over the first year of diagnosis.

Puolakka *et al.* [9] assessed the impact of the Stanford Health Questionnaire (HAQ) index on loss of productivity in early DMARD-naïve RA patients in the Finnish RA Combination Therapy (FIN-RACo) open-label extension clinical trial in Finland. Patients were randomized to either a combination of three DMARDs (SSZ, MTX and HCQ) and prednisolone, or a single DMARD with or without prednisolone [9] for 2 years and were followed up for 5 years. The mean disease duration across the four HAQ groups was between 8 and 11 months. In the overall cohort and over 5 years, the annual mean loss of productivity per patient was €8344 (95% CI 6516, 10 480) by the HCA and €1928 (95% CI 1567, 2298) by the friction cost approach (FCA). Functional capacity was assessed by HAQ at baseline and 6 months. The HAQ score after 6 months of treatment, but not the level of HAQ at baseline, predicted productivity costs in the overall cohort. Over 5 years, the top HAQ quartile had the highest work disability days per year [mean 273 days (95% CI 194, 328)], compared with the lowest HAQ quartile [mean 34 days (95% CI 5, 145)]. After adjusting for PPP and CPI 2021, the annual mean loss of productivity in USD 2021 in the top quartile group was $40 116 by the HCA method and $6125 by the FCA method. No analysis was performed to assess the impact of disease duration on costs in the overall cohort or by HAQ groups.

Verstappen *et al.* [10] assessed the total annual direct costs over different follow-up periods after first DMARD in Dutch patients with RA and identified sociodemographic, clinical and psychological predictors of high costs in two RCTs. Patients in the first RCT were randomized into one of four treatment arms [pyramid (NSAID followed by a DMARD for treatment failure), i.m. gold, MTX or HCQ]. Patients from the second RCT were randomized into intensive *vs* conventional MTX regimes. In this study, costs data were classified into three groups with increasing follow-up duration after diagnosis (0 to ≤2 years, 2 to ≤6 years and 6 to ≤10 years). In addition, RA patients with disease duration ≥10 years from the Utrecht RA Cohort study group were included to capture costs data for patients with longstanding RA. There was a significant difference in annual direct costs between the four groups. The median annual direct costs per patient showed a U-shaped distribution, i.e. costs were high for patients with follow-up duration 0 to ≤2 years (€2923) and reduced after 2–6 years (€1967), but increased again for ≥10 years follow-up duration (€3778). Data from the group with the shortest follow-up duration were extracted for Table 1. Functional disability (HAQ) was the most important variable associated with high costs after adjusting for sociodemographic, clinical and psychological variables. After adjusting for PPP and CPI 2021, the annual mean (median) of total direct costs per patient in USD 2021 was $14 613 ($8159). The annual direct costs of early RA follow a U-shaped distribution over 10 years following the start of DMARDs. No analysis was performed to assess the impact of disease duration at baseline on costs in the overall cohort.

Merkesdal *et al.* [11] reported the magnitude of indirect costs, changes within cost components and the correlation between changes in cost and social, clinical and occupational variables within first 3 years for DMARD-naïve RA patients in a multicentre observational study in Germany. The average indirect cost in early RA at the 24-month follow-up was high; $11 750 per person-year (US dollars for the period 1994–1996), which related to 126 days of loss of productivity. Loss of productivity owing to sick leave accounted for 84% of overall loss of productivity (sick leave, work disability and other work loss) between the onset of disease and the end of the first year after study enrolment, compared with only 25% at the end of the second year of the study enrolment [11]. After adjusting for PPP and CPI 2021, the mean costs associated with total sick leave, work disability and other work losses in USD 2021 were $20 180 after 12 months of follow-up and $18 848 per person per year at the 24-month follow-up time point. The relationship between disease duration and loss of productivity was not reported.

Newhall-Perry *et al.* [13] assessed the direct and indirect costs of seropositive RA patients 6 months before diagnosis in a longitudinal observational study at rheumatology centres in the western USA and Mexico. All patients were DMARD-naïve and had clinically active disease, with at least nine tender and six swollen joints and a positive RF. Patients were classified as disease duration of <6 months (*n* = 87) and ≥6 months (*n* = 63). At baseline, the mean total direct costs and indirect costs of RA 6 months before diagnosis were $200 per month and $281 per month in 1994 USD, respectively. The total direct costs of RA [mean (s.d. )] 6 months before diagnosis in patients with disease duration <6 months compared with ≥6 months were $240/month ± $285 and $144/month ± $149, *P* < 0.001, respectively. Likewise, indirect costs were higher in patients with a disease duration <6 months as opposed to ≥6 months ($348/month ± $567 *vs* $188/month ± $506; *P* < 0.005) at baseline. After adjusting for PPP and CPI 2021, the annual mean total direct and indirect costs 6 months before diagnosis per person in USD 2021 were $12 663 for <6 months and $7174 for ≥6 months groups. Overall, annual direct and indirect costs 6 months before RA diagnosis were higher in patients with shorter symptom duration (<6 months).

van Jaarsveld *et al.* [12] assessed the annual direct cost related to RA during the first 6 years and identified socioeconomic and clinical determinants of these costs in an RCT conducted in the Netherlands. Patients were recruited between 1990 and 1996, and cost questionnaires were sent to those not lost to follow-up in April 1996. Mean annual direct costs by follow-up duration (year 1–6) followed a U-shaped distribution, as follows: Dutch florin (Dfl.) 14 455/patient in year 1; Dfl.13 800/patient in year 2; Dfl. 9457/patient in year 3; Dfl. 6233/patient in year 4; Dfl. 13 005/patient in year 5; and Dfl. 11 158/patient in year 6. After adjusting for PPP and CPI 2021, total direct costs per patient (mean) in USD 2021 were $24 094 after 1 year follow-up duration. The annual direct costs of early RA showed a U-shaped distribution over 6 years following the start of DMARDs. No analysis was performed to assess the impact of disease duration at baseline on costs in the overall cohort.

A number of studies were excluded because study participants could receive at least one DMARD before study enrolment [18–21]. Tables 3 and 4 summarize the direct and indirect costs in USD 2021, respectively, and outcomes by increasing symptom or disease duration.

**Table 3.** Direct costs in USD 2021, symptom duration and outcomes according to increasing symptom or disease duration

Author, country, year	Symptom or disease duration	Symptom or disease duration (days)	Currency in USD 2021	Outcome
Verhoeven *et al.* [15] The Netherlands, 2021	Symptom duration	Median: TCZ+MTX 24.5 TCZ 25.5 MTX 27.0	Mean: TCZ + MTX 15 546 TCZ 18 350 MTX 17 840	Direct health-care-related costs by treatment strategy group, per patient per year

Luurssen-Masurel *et al.* [14] The Netherlands, 2021	Symptom duration	Median: 134	Mean: iMTX 3456 iHCQ 2839 iGC 4079	Healthcare costs by treatment strategy group, patient per year

Verstappen *et al.* [10] Netherlands, 2004	Disease duration	Mean: 329	Mean: 14 613 Median: 8159	Total direct costs per patient per year

van Jaarsveld *et al.* [12] The Netherlands, 1998	Disease duration	Inclusion criteria: 0–365	Mean: 16 472	Direct medical cost per person per year, per patient

Syngle *et al.* [16] India, 2017	Disease duration	Mean: 2117	Average: 1008	Direct medical cost per patient per year

iGC: initial treatment strategy with glucocorticoids; iHCQ: initial treatment strategy with HCQ; iMTX: initial treatment strategy with MTX; TCZ: tocilizumab.

**Table 4.** Indirect costs in USD 2021, symptom duration and outcomes according to increasing symptom or disease duration

Author, country, year	Symptom or disease duration	Symptom or disease duration (days)	Currency in USD 2021	Outcome
Merkesdal *et al.* [11] Germany, 2001	Disease duration	Mean: 213	Mean: Time 0–time 2: 20 180 Time 2–time 3: 15 865 Time 0–time 3: 18 848	Loss of productivity costs: total sick leave, work disability and other work loss

Luurssen-Masurel *et al.* [14] The Netherlands, 2021	Symptom duration	Median: 134	Mean: iMTX 11 031 iHCQ 12 149 iGC 9967	Total productivity costs by treatment strategy group

Verhoeven *et al.* [15] The Netherlands, 2021	Symptom duration	Median: TCZ+MTX 24.5 TCZ 25.5 MTX 27.0	Human capital approach: TCZ + MTX 17 076 TCZ 14 272 MTX 16 566 Friction cost approach: TCZ + MTX 6371 TCZ 5862 MTX 6371	Loss of productivity costs loss using human capital approach and friction cost approach by treatment strategy group

Puolakka *et al.* [9] Finland, 2009	Disease duration HAQ group 1	Mean: 335	Mean: HCA 736 FCA 590	Loss of productivity cost by human capital approach and friction cost approach by HAQ group
	HAQ group 2	243	HCA 4523 FCA 2275	
	HAQ group 3	243	HCA 20 191 FCA 4101	
	HAQ group 4	304	HCA 40 116 FCA 6125	

iGC: initial treatment strategy with glucocorticoids; iHCQ: initial treatment strategy with HCQ; iMTX: initial treatment strategy with MTX; TCZ: tocilizumab; time 0: onset of disease; time 2: reassessment at 12 months following baseline assessment; time 3: reassessment at 24 months following baseline assessment.

## Discussion

This study highlighted several interesting findings. Firstly, two studies reported a U-shaped distribution of costs over disease duration following an RA diagnosis. Total costs were high during the initial years, slightly lower thereafter, then high again for a disease duration of ≥5 years [12] and >10 years [10]. This indicates that costs are not a linear function of disease duration.

Secondly, functional disability was a predictor of productivity costs in three studies [9, 10, 12]. In one study, patients from the highest HAQ group had the highest work disability days per year, hence the highest costs for loss of productivity [9]. This finding is highly relevant. It supports the hypothesis that aggressive early treatment can reduce costs in the longer term, because those treated earlier are less likely to have a higher level of disability, which then translates to a lower loss of productivity costs in the long term.

One study reported that the annual direct and indirect costs 6 months before diagnosis were higher in those with a symptom duration of <6 months before the start of DMARD therapy compared with those with a symptom duration ≥6 months [13]. In contrast, another study reported that longer symptom duration before diagnosis (>180 days) was associated with lower health-care utilization over the first year of diagnosis [8]. The contrasting trend between the two studies can be explained by the difference in the timing of when the health economic outcomes were recorded. Health-care utilization over the first year following RA diagnosis was recorded in the latter study; however, costs before RA diagnosis were recorded in the former study.

**Figure 2.**
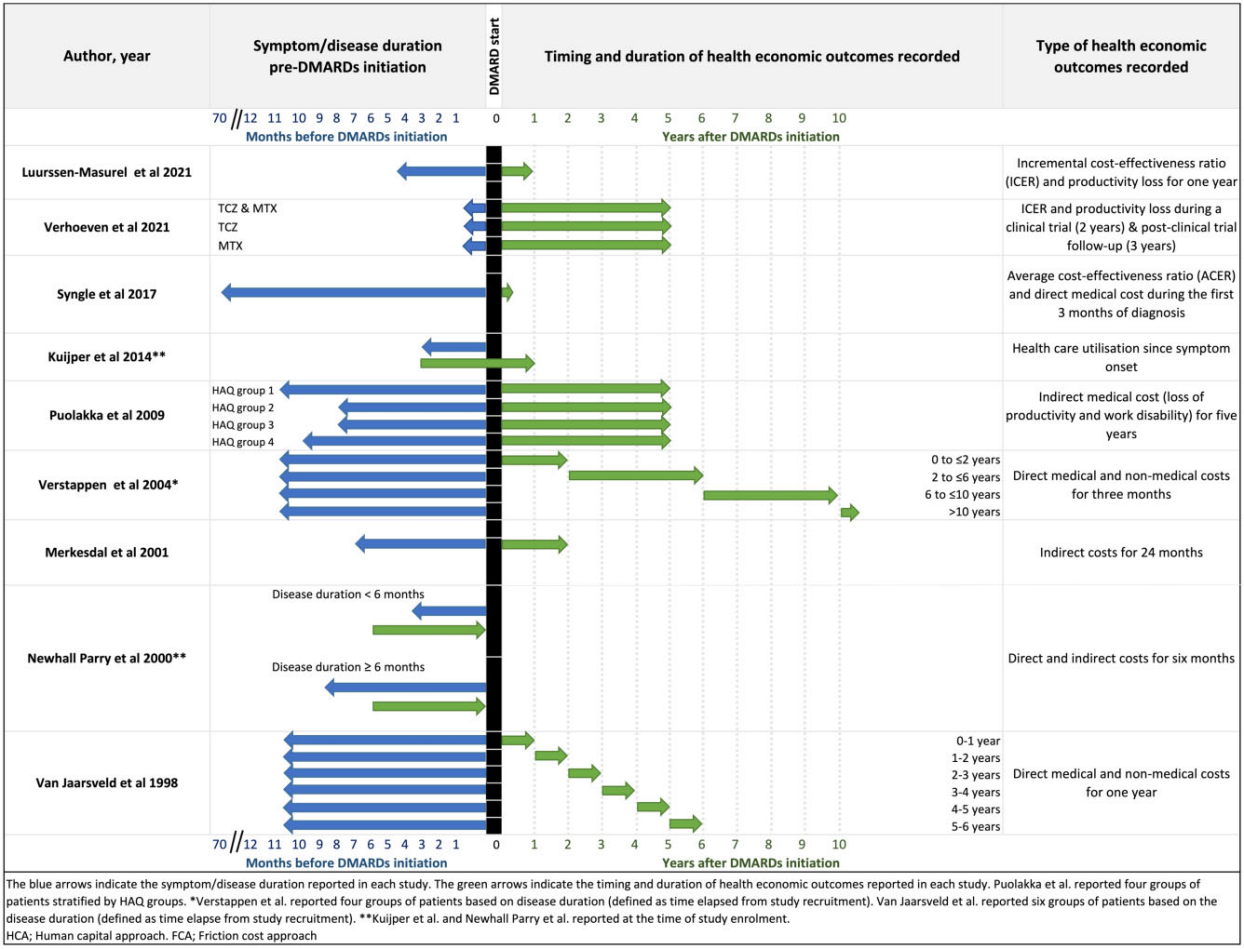
Timing and duration for which the respective health economic outcomes are reported and the symptom duration before DMARD initiation. The blue arrows indicate the symptom/disease duration reported in each study. The green arrows indicate the timing and duration of health economic outcomes reported in each study. Puolakka *et al*. [9]  reported six groups of patients stratified by HAQ groups. ^a^Verstappen *et al*. [10]  reported four groups of patients based on disease duration (defined as the time elapsed from study recruitment). Van Jaarsveld *et al*. [12]  reported six groups of patients based on disease duration (defined as the time elapsed from study recruitment). ^b^Kuijper *et al.* [8] and Newhall-Perry *et al*. [13]  reported disease duration at the time of study enrolment. FCA: friction cost approach; HCA: human capital approach

In this review, we could not delineate the aggregated-level data related to the relationship between symptom/disease/diagnosis duration and cost categories owing to the heterogeneity of the following factors: timing and duration of data collection regarding resources and costs; type of resources/cost-categories reported; and inconsistency in reported disease, symptom or diagnosis duration (Fig. 2). Moreover, the duration of cost data recorded (i.e. 6 months *vs* 6 years) also differed across studies (Fig. 2).

Before the era of early treatment, RA costs were related to established disease. Patients had more frequent hospitalization [22] and more frequent joint replacement than the general population [23], and a majority were unable to work. The early introduction of biological and targeted synthetic DMARD therapy has resulted in high costs of medications [23]. However, high drug cost can potentially be offset in the long term, at least in part, by reducing disease-related costs (e.g. loss of productivity owing to work disability, hospitalization and joint surgery). In addition, patients treated early were more likely to achieve DMARD-free remission [1]. Therefore, this would reduce the proportion of patients on long-term DMARDs [24].

Clear definitions of RA onset and duration have been proposed [25], because reporting in clinical studies is currently heterogeneous [25]. RA duration can be timed from the following points: onset of RA symptoms; onset of joint swelling; when RA classification criteria were first fulfilled; or the time of RA diagnosis. Using a clearly defined onset will allow meaningful comparison of clinical outcomes and health economic outcomes between early RA studies.

A strength of this review is the broad range of health economic outcomes and types of health economic studies that were included. Both direct and indirect costs, and cost-of-illness and cost-utility studies were within the scope of this review. Observational and clinical trials were also included.

However, only a small number of studies fulfilled our strict inclusion criteria. In addition, studies that enrolled patients who had recently been treated with DMARDs before study recruitment were not included in this review. Furthermore, meta-analyses/regression were not possible owing to the different types of health economic outcomes reported.

This review is the first to highlight a vital evidence gap in early arthritis: what is the financial consequence of diagnosing and treating patients with RA during the early disease phase? Health economic modelling with carefully defined symptom duration, resource utilization, treatment and long-term productivity costs is vital to address this important question.

## Supplementary Material

rkad040_Supplementary_Data

## Data Availability

The data underlying this article will be shared on reasonable request to the corresponding author.
