# Supplementary material for: Is symptom duration before DMARD therapy a determinant of direct and indirect costs in DMARD-naïve RA patients? A systematic review
Source: Rheumatol Adv Pract. 2023 Apr 13;7(2):rkad040. doi: 10.1093/rap/rkad040 (PMC10185403; doi:10.1093/rap/rkad040)
Supplement: rkad040_Supplementary_Data [file rkad040_supplementary_data.docx]

Supplementary data

Contents

[Supplementary Data S1. Methods 2](#_Toc129610367)

[Supplementary Data S2. Search terms and strategy 4](#_Toc129610368)

[Supplementary Table S1. Patients’ socio-demographic, disease characteristics and inclusion and exclusion criteria for clinical studies. 8](#_Toc129610369)

[Supplementary Table S2. Study characteristics, health economic outcomes and annual costs in local currency and US Dollars 2021 14](#_Toc129610370)

[Supplementary Data S3. Quality assessment 28](#_Toc129610371)

[Supplementary Table S3. Quality assessment based on modified checklist described by Drummond and Jefferson (3) and STROBE (2) checklists. 29](#_Toc129610372)

# Supplementary Data S1. Methods

**Protocol and Registration**

The study protocol was developed based on Preferred Reporting Items for Systematic Review and Meta-Analysis guidelines (PRISMA)(1). The protocol for this systematic review was registered on the International Prospective Register of Systematic Reviews (PROSPERO 2017 CRD42017077593) and can be accessed at https://www.crd.york.ac.uk/prospero/display_record.php?ID=CRD42017077593.

**Study identification/Search Strategy**

PubMed, EMBASE, CINAHL and Medline electronic databases were searched initially on 31 August 2017. Three further searches were carried out on 18 October 2018,23 December 2021 and 25 January 2023, to capture all relevant articles prior to completion of the systematic review. The date range for first search was from database inception to 31 August 2017; second search from 1 September 2017 to 18 October 2018, third search from 1 October 2018 to 23 December 2021, and last search from 24 December 2021 to 25 January 2023 All systematic searches were conducted using the search terms and strategy presented in supplementary figure S2. All systematic searches were conducted using the same terms on the same databases.

Studies relating to RA, DMARD therapy and direct and indirect costs with no language restriction were included. Results were de-duplicated, and the title and abstract of each paper were made available for subsequent eligibility assessment. Additional records were identified through independent manual database searching, external sources and reference scanning of relevant retrieved full-text articles.

**Study selection**

Study inclusion criteria were studies with i) patients aged ≥ 18 years fulfilling 1987 American College of Rheumatology (ACR) or 2010 ACR/European Alliance of Associations for Rheumatology (EULAR) RA classification criteria, ii) DMARD-naïve patients, iii) symptom or disease duration reported, iv) cross-sectional and longitudinal study design, and v) health economic outcomes reported as costs or resource utilisation.

Studies excluded were (1) studies of non-RA inflammatory arthritides; (2) conference abstracts, systematic reviews or review articles; (3) studies with no reported symptom or disease duration and (4) studies with no reported health economic outcomes. Two authors (IS, RS) independently reviewed the title and abstract of the search results for suitability. Subsequently, the full texts of eligible articles were retrieved and independently reviewed (IS, RS) for eligibility for data extraction, with reasons for exclusion recorded. Discrepancies were resolved first by consensus or through a third reviewer (ABo).

**Data extraction**

Two authors (IS, RS) independently extracted these data from full manuscripts of eligible studies:

1) Study characteristics (country, study design, study year, objectives, source of funding, the source for sampling, sample size, inclusion/exclusion criteria and specifically disease duration at inclusion).

2) Potential determinants of RA costs (sex, age, ethnicity, disease/symptom duration, autoantibody status, inflammatory markers, body mass index, disease activity score, smoking, functional status and socio-economic factors).

3) Sources of (i) resource utilisation and (ii) costs, and

4) Health economic outcomes (direct medical resources/costs, direct non-medical costs and indirect costs.)

Discrepancies were resolved first by consensus or through a third reviewer (ABo).

**Quality assessment**

Two authors (IS, RS) independently rated each article using two quality assessment tools; the Strengthening The Reporting of Observational Studies in Epidemiology (STROBE) checklist (2) and a modified checklist described by Drummond and Jefferson, which incorporated items relevant to these health economic studies (3). Each item in both checklists was scored as ‘yes’, ‘no’, ‘partly’ or ‘not applicable’. Discrepancies were resolved by consensus or through a third reviewer (ABo).

**Data synthesis and statistical analysis**

A meta-analysis or meta-regression on the association between disease/symptom duration and costs could not be assessed due to the low number of available studies and clinical and methodological heterogeneity, especially in assessing and reporting of health economic outcomes. Cost data per patient per year for the reported duration in the study were recorded and summarised in a unifying currency of US Dollars 2021 after adjusting for Purchasing Power Parity (PPP) and Consumer Price Index (CPI) 2021 (4, 5).

# **Supplementary Data S2.** Search terms and strategy

# Database (Search term) Results

1 EMBASE exp "RHEUMATOID ARTHRITIS"/ 180492

2 EMBASE (rheumatoid arthritis).ti,ab 132729

3 EMBASE (1 OR 2) 196948

4 EMBASE (direct).ti,ab 803646

5 EMBASE (indirect).ti,ab (180935)

6 EMBASE (illness).ti,ab (257944)

7 EMBASE (societal).ti,ab (23079)

8 EMBASE (4 OR 5 OR 6 OR 7) (1185606)

9 EMBASE (cost*).ti,ab (642342)

10 EMBASE (8 AND 9) (65392)

11 EMBASE (absenteeism).ti,ab (6655)

12 EMBASE ("work productivity").ti,ab (2828)

13 EMBASE ("economic burden").ti,ab (12187)

14 EMBASE ("income loss").ti,ab (226)

15 EMBASE ("work disability").ti,ab (2176)

16 EMBASE ("economic evaluation").ti,ab (10097)

17 EMBASE (10 OR 11 OR 12 OR 13 OR 14 OR 15 OR 16) (91053)

18 EMBASE (3 AND 17) 1986

19 EMBASE (determin*).ti,ab 3827926

20 EMBASE (18 AND 19) 397

21 Medline exp "ARTHRITIS, RHEUMATOID"/ 102713

22 Medline (rheumatoid arthritis).ti,ab 92994

23 Medline (21 OR 22) 132571

24 Medline (direct).ti,ab 656943

25 Medline (indirect).ti,ab 151343

26 Medline (illness).ti,ab 190097

27 Medline (societal).ti,ab 17778

28 Medline (24 OR 25 OR 26 OR 27) 953870

29 Medline (cost*).ti,ab 460678

30 Medline (28 AND 29) 43575

31 Medline (absenteeism).ti,ab 4677

32 Medline ("work productivity").ti,ab 1264

33 Medline ("economic burden").ti,ab 7203

34 Medline ("income loss").ti,ab 158

35 Medline ("work disability").ti,ab 1703

36 Medline ("economic evaluation").ti,ab 7110

37 Medline (30 OR 31 OR 32 OR 33 OR 34 OR 35 OR 36) 60879

38 Medline (23 AND 37) 883

39 Medline (determin*).ti,ab 2992627

40 Medline (38 AND 39) 162

41 CINAHL exp "ARTHRITIS, RHEUMATOID"/ 14593

42 CINAHL (rheumatoid arthritis).ti,ab 9938

43 CINAHL (41 OR 42) 16719

44 CINAHL (direct).ti,ab 39763

45 CINAHL (indirect).ti,ab 9464

46 CINAHL (illness).ti,ab 274674

47 CINAHL (societal).ti,ab 5236

48 CINAHL (44 OR 45 OR 46 OR 47) 315522

49 CINAHL (cost*).ti,ab 85761

50 CINAHL (48 AND 49) 18134

51 CINAHL (absenteeism).ti,ab 1450

52 CINAHL ("work productivity").ti,ab 331

53 CINAHL ("economic burden").ti,ab 1315

54 CINAHL ("income loss").ti,ab 27

55 CINAHL ("work disability").ti,ab 508

56 CINAHL ("economic burden").ti,ab 1315

57 CINAHL (50 OR 51 OR 52 OR 53 OR 54 OR 55 OR 56) 20983

58 CINAHL (43 AND 57) 374

59 CINAHL (determin*).ti,ab 230990

60 CINAHL (58 AND 59) 63

61 PubMed (rheumatoid arthritis).ti,ab 133956

62 PubMed (direct).ti,ab 662838

63 PubMed (indirect).ti,ab 164631

64 PubMed (illness).ti,ab 427369

65 PubMed (societal).ti,ab 18269

66 PubMed (62 OR 63 OR 64 OR 65) 1201349

67 PubMed (cost*).ti,ab 517241

68 PubMed (66 AND 67) 63987

69 PubMed (absenteeism).ti,ab 10719

70 PubMed ("work productivity").ti,ab 1274

71 PubMed ("economic burden").ti,ab 7299

72 PubMed ("income loss").ti,ab 149

73 PubMed ("work disability").ti,ab 1739

74 PubMed ("economic burden").ti,ab 7299

75 PubMed ("economic evaluation").ti,ab 7662

76 PubMed (68 OR 69 OR 70 OR 71 OR 72 OR 73 OR 74 OR 75) 85925

77 PubMed (61 AND 76) 1204

78 PubMed (determin*).ti,ab 3080123

79 PubMed (77 AND 78) 219

80 EMBASE ((direct OR indirect) AND cost).ti,ab 34795

81 EMBASE exp "DISEASE MODIFYING ANTIRHEUMATIC DRUG"/ 11109

82 EMBASE (DMARD).ti,ab 5606

83 EMBASE (81 OR 82) 14556

84 EMBASE (3 AND 80 AND 83) 142

85 Medline ((direct OR indirect) AND cost).ti,ab 22173

86 Medline exp "ANTIRHEUMATIC AGENTS"/ 403640

87 Medline ("disease modifying antirheumatic drug").ti,ab 873

88 Medline (DMARD).ti,ab 1936

89 Medline (86 OR 87 OR 88) 404206

90 Medline (23 AND 85 AND 89) 138

91 CINAHL (41 OR 42) 16731

92 CINAHL ((direct OR indirect) AND cost).ti,ab 4196

93 CINAHL ("disease modifying antirheumatic drug").ti,ab 224

94 CINAHL exp "ANTIRHEUMATIC AGENTS"/ 32530

95 CINAHL (DMARD).ti,ab 471

96 CINAHL (93 OR 94 OR 95) 32681

97 CINAHL (91 AND 92 AND 96) 27

98 PubMed ("disease modifying antirheumatic drug").ti,ab 889

99 PubMed (DMARD).ti,ab 421188

100 PubMed (98 OR 99) 421300

101 PubMed ((direct OR indirect) AND cost).ti,ab 31741

102 PubMed (rheumatoid arthritis).ti,ab 133996

103 PubMed (100 AND 101 AND 102) 169

The data above show the search terms and search strategy used for the three systematic searches. The date range for first search was from database inception to 31^st^ August 2017; second search was from 1^st^ September to 18^th^ October 2018, third search was from 19^th^ October 2018 to 23^rd^ December 2021, and final search was from 24^th^ December 2021 to 25^th^ January 2023. All systematic searches were conducted using the same terms on the same databases.

Supplementary Figure S3.

| Supplementary Table S1. Patients’ socio-demographic, disease characteristics and inclusion and exclusion criteria for clinical studies. | | | | | |
| --- | --- | --- | --- | --- | --- |
| **Author** | **Sociodemographic**  **Place,**  **Number of patients,**  **Ethnicity**  **Employment**  **Level of Education**  **Marital status** | | **Disease characteristics**  **Disease activity score**  **CRP, mg/dl [mean (SD)]**  **ESR, mm/H [mean (SD)]**  **Functional status**  **Smoking, Co-morbidities**  **BMI [mean (SD)]** | **Study inclusion and exclusion criteria** |  |
| **Luurssen-Masurel et al. (2021) (14)** | Rotterdam, Netherlands  **N total =**116  MTX: 44  HCQ: 35  GC: 37  **Paid work, n (%)**  MTX: 17 (44)  HCQ: 21 (66)  GC: 20 (61)  **Retired, n (%)**  **MTX:** 5 (11)  **HCQ:** 3 (9)  **GC:** 4(11) | | **DAS, mean (SD):**  MTX: 3.51 (0.92)  HCQ: 3.00(0.85)  GC: 3.57 (0.94)  **SJC44, median (IQR)**  MTX: 9 (6-13)  HCQ: 6 (2-10)  GC: 8 (4-15)  **Erosive disease, n (%)**  MTX: 1(2)  HCQ: 0(0)  GC: 0(0) | **Participants in the tREACH clinical trial with these criteria:**   1. intermediate probability of developing persistent arthritis based on the Visser prediction model 2. autoantibody negative (both RF and ACPA negative). 3. fulfil RA 2010 at baseline   **tREACH clinical trial inclusion criteria:**   1. age ≥18 years, 2. arthritis in one or more joint(s) 3. symptom duration <1 year   **tREACH clinical trial exclusion criteria:**  1. Diagnosed with  a. a crystal arthropathy,  b. a (post-)infectious arthritis, or  c. an autoimmune disorder other than RA  2. Previous DMARD therapy or glucocorticoid usage  3. Contra-indications for initial study medication, namely:  a. chronic liver disease  b. excessive alcohol and drug use  c. pregnancy (wish)  d. leucopenia <3.0 × 109/l  e. thrombocytopenia <150 × 109/l  f. aspartate aminotransferase/ alanine aminotransferase more than two times the upper normal value  g. creatinine level >150 µmol/l. |  |
| **Verhoeven et al. (2021) 15** | Netherlands  **N**=317  TCZ+MTX: 106  TCZ:103  MTX:108  **Employed, n(%)**  TCZ+MTX: 85 (80)  TCZ: 79 (77)  MTX: 87 (81)  **Working hours per week, mean (SD)**  TCZ+MTX: 24.0 (15.3)  TCZ: 22.1 (15.5)  MTX: 24.6 (15.8) | | **DAS28, mean (SD)**  TCZ+MTX: 5.2 (1.1)  TCZ: 5.3 (1.1)  MTX: 5.1 (1.2)  **HAQ, mean (SD)**  TCZ+MTX: 1.1 (0.67)  TCZ: 1.3 (0.66)  MTX: 1.1 (0.59)  **RF, n (%)**  TCZ+MTX: 75 (71)  TCZ: 68 (66)  MTX: 86 (80)  **Anti-CCP, n (%)**  TCZ+MTX: 72 (68)  TCZ: 67 (65)  MTX: 84 (78) | **Inclusion criteria:**   1. DMARD-naive 2. 18 years or older 3. Fufill the classification criteria for rheumatoid arthritis (1987 ACR or 2010 ACR/EULAR) 4. diagnosed within 1 year before inclusion 5. disease activity score of 28 joints (DAS28)of at least 2.6, indicating presence of disease activity.   **Exclusion criteria:**   1. contraindications according to standard of care for patients with RA for starting a biological DMARD or methotrexate, such as uncontrolled serious co-morbidities, active or recurrent infections, malignancy and drug or substance misuse. 2. Patients with another autoimmune or inflammatory rheumatic disease 3. Patients who are on oral glucocorticoids within 6 weeks before baseline 4. Patient who are unable to comply with the requirements of the study protocol. |  |
| **Syngle et al. 2017 16** | Chandigarh, India.  **N**=98 | | **DAS-28** ‡‡: 6.07 +- 1.33  **CRP**: 15.48 +- 11.74  **ESR**: 36.1+-15.2  **HAQ - Di** ‡‡ : 1.43+-0.71  **BMI**: 23.66 (4.49)  **Smoking** : 0 | **Inclusion:**  1. Aged > 18 years  2. Symptom duration > six months  3. DMARD-naïve  4. RA 2010 criteria  **Exclusion:**  1. Chronic liver and renal disease.  2. Untreated infection.  3.Leukopaenia/pancytopaenia  4. G6PD deficiency |  |
| **Kuijper et al. 2014 (6)** | Rotterdam, Netherlands.  **N**=244^§§^  **Dutch**^^: 77% (N=229)  Paid Employment†††: 55%  Level of education††:   1. Low = 63% 2. Intermediate =24% 3. High = 13%   Marital status:  Living alone†††:16% | | **RADAI*** =3.3 (0-9.5)***  **HAQ***: 1 (0-2.9) ^^  **Number of comorbidities**† (%)   1. None = 31% 2. 1 = 33% 3. 2 = 18% 4. 3+ = 13%   **BMI**: 26.4 (4.6)** | **Inclusion:** Had either or both of:  1. Synovitis in one joint on clinical examination.  2. ^#^Pain, stiffness, or loss of function, in at least two joints accompanied by at least two of the following criteria: morning stiffness of greater than one hour, unable to make a fist in the morning, pain when shaking someone hands, pins and needles in the fingers, difficulties wearing rings or shoes, family history of RA and unexplained fatigue lasting less than a year.  # Results were classified as arthralgia and RA patients. 87.3% of RA patients fulfilled RA 1987 and/or 2010 criteria. **Data for RA patients were extracted for this review.**  **Exclusion:**  1. Symptom duration > 12 months.  2. Symptom due to trauma or overuse. |  |
| **Puolakka et al. 2009^§^ (7)** | Finland.  **N**=159 | ***HAQ Category 1***  **N**=13  **Years of education**‡‡: 12 (4) | **Tender Joint Count**‡‡: 13(4)  **Swollen Joint Count**‡‡ : 13(3)  **VAS-pain**‡‡: 29 (26)  **Patients global assessment**‡‡: 27(25)  **Physician global assessment**‡‡: 35(17)  **ESR**: 27 (14)  **HAQ** ^‡‡^: 0 | **Inclusion:**  1. Aged 18 - 65 years  2. Disease duration< 2 years  3. RA 1987 criteria  4. Active disease with ≥ 3 swollen joints and at least three of the following:  a) ESR ≥ 28 mm/h  b) CRP > 19 mg/L  c) morning stiffness ≥ 29 min  d) > 5 swollen joints and > ten tender joints.  **Exclusion criteria:**  1. Previous DMARDs.  2. Previous use of glucocorticoid in 2 weeks  3. Serious comorbidities  4. Suspected inability to comply with protocol  5. Hypersensitivity to study medication  6. History of cancer  7. Pregnancy  8. Women of childbearing age without reliable contraception. |  |
|  |  | ***HAQ Category 2***  **N**=65  **Years of education**‡‡: 11(4) | **Tender Joint Count**‡‡: 17(8)  **Swollen Joint Count**‡‡: 13(7)  **VAS-pain**‡‡: 44 (22)  **Patients global assessment**‡‡: 43 (18)  **Physician global assessment**‡‡: 44(17)  **ESR**: 37 (23)  **HAQ** ^‡‡^: 0.7 (0.4) |  |  |
|  |  | ***HAQ Category 3***  **N**=65  **Years of education**‡‡: 11(3) | **Tender Joint Count**‡‡: 21 (10)  **Swollen Joint Count**‡‡: 14 (7)  **VAS-pain**‡‡:50 (23)  **Patients global assessment**‡‡: 52 (22)  **Physician global assessment**‡‡: 49 (19)  **ESR**: 39 (23)  **HAQ** ^‡‡^: 1.0 (0.7) |  |  |
|  |  | ***HAQ Category 4***  **N**=16  **Years of education**‡‡: 11(4) | **Tender Joint Count**‡‡: 23(10)  **Swollen Joint Count**‡‡: 14(5)  **VAS-pain**‡‡: 65 (20)  **Patients global assessment**‡‡: 64 (21)  **Physician global assessment**‡‡: 58 (19)  **ESR:** 45 (25)  **HAQ** ‡‡: 1.5 (0.5) |  |  |
| **Verstappen et al. 2004 10** | Utrecht, Netherlands  **N**= 509 | | **VAS-RA**, mm (SD): 67 (24)  **Marital Status:**  i. Married 62 (65%)  ii. Unmarried 14 (15%)  iii.Divorced 10 (10%)  iv.Widowed 10 (10%)  **Level of education‡:**  i. Low: <high vocational /university = 86%  ii. High: ≥high vocational /university = 14% | **Inclusion:**  1.Disease duration ≤ 1 year  2.RA 1987 criteria  **Exclusion:**  1. Aged < 17 years  2. Comorbidities that affects study treatment.  3.Previous or current treatment with SAARDs, glucocorticosteroids, cytotoxic or immunosuppressive therapy,  4. Possibility of pregnancy or breast feeding  5.Psychiatric or mental illness that affects adherence to study protocol |  |
| **Merkesdal et al 2001 11** | Germany  N= 133  **Employment variables:**   - Blue-collar worker: n=44(34%). - White-collar worker: n=86(66%). - Working hours/weak, mean (SEM): 34 (0.9). - Gross income, mean (SEM) $/month 2090 (1170). | | **Tender Joint Count‡‡:** not stated.  **Swollen Joint Count, mean (SEM) :** 11 (0.6).  **Patient VAS-pain (0-3) ‡‡:** not stated.  **Patients global disease activity (VAS; 0-3) ‡‡:** not stated:  **Physician global assessment‡‡ (VAS; 0-100):** not stated.  **ESR, mean (SER): 30 (2.2)** | **Inclusion:**   1. Fulfilled 1987 RA criteria. 2. < 12 months from onset of joint swelling.   **Exclusion:**   1. Patients who were not employed. 2. Patients lost to follow-up. 3. Aged >65 4. Received disability payments for non-RA reasons |  |
| **Newhall-Perry et al.2000 13** | Western US and Mexico.  **N:** 150  **White:** 78%  **Employment:**   - Employed : 52%   **Education level in years:** mean (SD) ; 12.8 (2.9).  **Marital status:**   - Married: 75% | | **Tender Joint Count‡‡:**  24.9 (13.5)  **Swollen Joint Count‡‡:** 20.6 (11.6)  **Patient VAS-pain (0-3) ‡‡:** 1.53 (0.77)  **Patients global disease activity (VAS; 0-3) ‡‡:** 1.3 (0.7)  **Physician global assessment‡‡ (VAS; 0-100) :** 50.7 (21.6)  **CRP:** 3.0 (3.9)  **ESR:** 41.2 (22.4)  **HAQ Disability Index (0-3) ‡‡:** 1.24 (0.70).  **With co-morbidities :** 54% | **Inclusion:**   1. RA 1987 criteria. 2. Clinically active disease defined by at least 9 tender and 6 swollen joints. 3. Positive RF 4. Disease duration of less than one year.   **Exclusion:**   1. >10 mg of prednisolone daily. 2. DMARD naïve 3. <16 years old. |  |
| **Van Jaarsveld et al. 1998 (8)** | Utrecht, Netherlands    N=363 | | **Radiographic damage:**  (% Sharp-score ≥ 1): 68%  **Presence of comorbidity** ≥ 1: 40% | **Inclusion:**  1. Disease duration ≤ 1 year  2. RA 1987 criteria  **Exclusion:**  1. Aged < 17 years  2. Comorbidities that affects study treatment  3. Previous or current treatment with SAARDs, glucocorticosteroids, or cytotoxic or immunosuppressive therapy,  4. Possibility of pregnancy or breast feeding  5.Psychiatric or mental illness that affect adherence to study protocol |  |

SAARDs (slow acting anti rheumatic drugs); *median (range); ‡‡ mean (SD); **N=219; ***N=255; ^^N=229; ‡N=96; †N=232; ††N=230; †††N= 231; RADAI: RA disease activity index; VAS: Visual Analogue Score. §Outcome data is split into 4 groups based on HAQ: Group1 (HAQ 0 at baseline and 6 m), Group 2 (HAQ>0 at baseline, 0 at 6m), Group 3 (HAQ≥0 at baseline, >0 but <1.0 at 6m), Group 4 (HAQ ≥0 at baseline, ≥1.0 at 6m) ; (radiological damage) range 0-448 sharp score ≥1 means at least one erosion or some joint space narrowing is visible). HAQ [value range 0-3; 3 worst score]. #The results were split between arthralgia and RA patients. 87.3% of RA patients fulfilled RA 1987 and/or 2010 criteria.

# Supplementary Table S2. Study characteristics, health economic outcomes and annual costs in local currency and US Dollars 2021

| **Author** | **Country**  **Number patients**  **Study duration**  **/year**  **Funding source** | **Objective**  **Study design**  **Study setting** | **Sample characteristics** | **Outcome**  **Frequency and recall assessment** | **Source of cost reference value or source of resource utilisation by categories**  **Study perspective** | **Results in local currency and year of assessment by cost categories** | **Results in local currency and year of assessment** | **Cost per person per year in USD 2021 after adjusting for purchasing power parity and consumer price index 2021 (OECD, 2021)(4, 5)** |
| --- | --- | --- | --- | --- | --- | --- | --- | --- |
| **Luurssen-Masurel (2021)14** | Rotterdam, Netherlands  N=116  **Recruitment period:** July 2007 until April 2011.  **Funding source:** tREACH trial was supported by Pfizer | **Objective:**  To assess cost-effectiveness of three different initial treatments in seronegative RA patients*  *Patients from the tREACH trial with intermediate probability of developing persistent arthritis (based on Visser score) fulfilled RA 2010 criteria, and were RF and ACPA negative at baseline. | **N**: 116  **F:** 81/116  **Symptom duration(median, IQR):** 134 days (95-205) days  **Age (average):** 54.8  **RF**: NA (all patients seronegative) | **Outcomes:** Incremental cost-effectiveness ratio (ICER) ratio between two of the three initial treatment strategies.  **Loss of productivity per year by:** Friction cost approach valued at age- and sex-dependent standard costs per hour.  **Time points of clinical assessment**:  3-monthly | **Medication costs:**  Dosages from patients’ case records, valued according to the Dutch college of health insurances.  **Medical consumption costs**  A. Dutch standard prices:   1. Inpatient day 2. Intensive care unit 3. Daycare treatment 4. Outpatient visit 5. Emergency room visit 6. Primary care physician 7. Paramedical care/physical therapy | **Currency:** Euros 2019  **Healthcare costs by treatment strategies group average per patient during 1 year of follow-up**  **Total mean (SD)**  **iMTX:** 2584 (2196)  **iHCQ:** 2123 (2172)  **iGC:** 3050 (3461)  **Productivity costs by treatment strategies group**  **Total in mean (SD)**  **iMTX:** 8249 (14,171)  **iHCQ:** 9085 (11,571)  **iGC:** 7453 (10,446) | **Total costs (healthcare and productivity costs) by treatment strategies group per year**  **Mean**  **iMTX:**10,832  **iHCQ:** 11,208  **iGC:** 10,502 | **Total costs (healthcare and productivity costs) by treatment strategy groups in USD 2021:**  **Mean**  iMTX: 14,485  iHCQ: 14,988  iGC: 14,044 |

|  |  | Patients with intermediate probability of developing persistent arthritis received one these initial treatments:   1. iMTX 25mg per week 2. iHCQ 400mg daily 3. oral iGCs, 10-week tapering course starting with 15mg daily, without any csDMARDs.   **Study setting:** Patient recruited from eight rheumatology centres in the Netherlands for the tREACH trial.  **Study design:** Cost-utility study alongside clinical trial of one-year duration. |  | **Recall of resource/costs assessment**:  3-monthly questionnaires on loss of productivity  6-monthly questionnaire on work time and employment | B. US standard prices  1. Complementary and alternative medicine costs (due to lack of dutch standard prices)  **Perspective:**  1.Partial societal  2. Healthcare |  | |  | |  | |
| --- | --- | --- | --- | --- | --- | --- | --- | --- | --- | --- | --- |
| **Verhoeven (2021)15** | Rotterdam, NetherlandsN= 317  **Recruitment period:** 13th January 2010 to 30th July 2012  **Funding source:** Roche Nederland BV | **Objective:**  To assess cost-effectiveness of initiating tocilizumab (TCZ) ± methotrexate (MTX) versus initiating MTX as treat-to-target treatment strategies over 5 years in early DMARD-naïve RA.  Treatment strategies were   1. TCZ+MTX 2. TCZ + placebo MTX 3. MTX + placebo TCZ   **Study design:** Cost-utility study in the context of a clinical trial (2 years) and post-clinical trial follow-up (3 years).  **Study setting:** 21 rheumatology outpatients clinic in the Netherlands | N: 317  **Female, n (%)**  **TCZ+MTX:** 65 (61)  **TCZ:** 78 (76)  **MTX**: 69 (64)  **Symptom duration, days, median (IQR)**  **TCZ+MTX:** 24.5 (16.0-41.5)  **TCZ:** 25.5 (18.0-45.0)  **MTX:** 27.0 (15.0-46.0)  **Age, years, median (IQR)**  **TCZ+MTX:** 53.0 (46.0 – 60.0)  **TCZ:** 55.0 (47.0-63.0)  **MTX:** 53.0 (44.5 – 62.0)  **RF, n (%)**  TCZ+MTX: 75 (71)  TCZ: 68 (66)  MTX: 86 (80) | **Outcomes:**   1. **Incremental cost-effectiveness ratios (ICER),** between two treatment strategies . 2. **Productivity loss costs** by human capital approach and friction cost approach.   **Time points of clinical assessment:** Monthly during the 2-year trial period, every 3 months during the first year of the post-trial follow-up, and every 6 months thereafter.  **Recall of resource/costs assessment:**  3-monthly healthcare resource use questionnaires   1. Other direct healthcare-related resource use e.g. physician visits, hospital admissions, non-RA medication 2. Indirect non-healthcare-related resource use: e.g. travel cost purchase of a stairlift)   **Productivity loss** Work participation questionnaire at baseline, 3, 6, 12 and 24 months during the trial, and yearly during post-trial follow-up. | **Medication costs:**  Medication use data was based on recorded dose and duration of (non-placebo) MTX and TCZ, and recorded use (yes/no) (NSAID).  Medication use was multiplied by national prices, separately for bDMARD, csDMARD, and NSAIDs in-line with Dutch costing manual  **Perspectives:**  1. healthcare perspective (including only healthcare cost)  2. partial societal perspective (including non-healthcare cost like costs related to productivity) | **Currency:** euros 2017 (€)  H **Costs** (€, rounded to the nearest hundreds) by treatment strategies group, means  **Direct healthcare-related costs**  **TCZ+MTX:** 6,100  **TCZ:** 7,200  **MTX:** 7,000  **Total productivity costs loss using human capital approach**  **TCZ+MTX:** 6,700  **TCZ:** 5,600  **MTX**: 6,500  **Total productivity loss costs using friction cost approach**  **TCZ+MTX:** 2,500  **TCZ:** 2,300  **MTX:** 2,500  **Indirect non-healthcare related costs**  **TCZ+MTX:** 1,100  **TCZ:** 1,600  **MTX:** 1,500 | | **Healthcare and productivity costs by treatment strategies group in euros 2017)**  **Mean per year, at end of year 1**  **Direct healthcare-related costs**  **TCZ+MTX:** 6,100  **TCZ:** 7,200  **MTX:** 7,000  **Total medication costs**  **TCZ + MTX:** 17,900  **TCZ:** 18,400  **MTX:** 4,400  **Total productivity costs loss using human capital approach**  **TCZ+MTX**: 6,700 **TCZ:** 5,600  **MTX:** 6,500  **Total productivity loss costs using friction cost approach**  **TCZ+MTX:** 2,500  **TCZ:** 2,300  **MTX:** 2,500  **Indirect non-healthcare related costs**  **TCZ+MTX:** 1,100  **TCZ:** 1,600  **MTX:** 1,500 | | **Healthcare and productivity costs by treatment strategies group in USD 2021**  **Mean per year, at end of year 1**  **Direct healthcare-related costs**  **TCZ + MTX:** 15,546  **TCZ:** 18,350  **MTX:** 17,840  **Total medication costs**  **TCZ + MTX:** 45,620  **TCZ:** 46,894  **MTX**: 11,214  **Total productivity costs loss using human capital approach**  **TCZ + MTX:** 17,076  **TCZ:** 14,272  **MTX:** 16566  **Total productivity loss costs using friction cost approach**  **TCZ + MTX:** 6371  **TCZ:** 5862  **MTX:** 6371  **Indirect non-healthcare related costs**  **TCZ + MTX:** 2803  **TCZ:** 4078  **MTX:** 3823 | |
| **Syngle et al. 2017 16** | Chandigarh, India  N=98  **Recruitme-nt period:**  Jan - Nov 2016  **Funding source:** Not stated | **Objective:**  To assess the cost and effects of synthetic DMARDs in treatment-naïve RA patients.  **Study design:**  Cost-utility study in the context of longitudinal observational study  **Study setting:**  One rheumatology outpatient clinic | **N**: 98 (F: 86%)  **Disease duration at inclusion:** 5.8 (SD 5.0 years)  **Age:**  47.8 (SD 12.3)  **RF**: 80% | **Outcome:**  Average cost-effectiveness ratio (ACER). Cost is measured in monetary value and the effectiveness of treatment measured as change in HAQ-DI.  Direct medical costs:   1. Medication 2. Monitoring 3. Consultation   **Time points of clinical assessment**: Baseline, 6 and 12 weeks.  **Recall of resource/costs assessment**: Three months | 1. **Medication costs**  Current Index of Medical Specialities  2. **Cost of Lab Investigations/ Radiology**  Government Multi Speciality Hospital, Chandigarh  3. **Physician Consultation charges**  Not stated  **Perspective:** Healthcare | **C Currency:** Indian Rupees 2017  **Direct medical costs**   1. Medication costs (average/month) 2. DMARDs = 398 3. Steroids = 136.3 4. NSAIDs = 16.66 5. Medicines to prevent Adverse drug reaction = 48.8 6. Monitoring Costs (average/month) 7. Lab Costs = 354 8. Radiology = 24.3 9. Ophthalmology = 5.97 10. Consultation Charges (average/month) 11. Doctors Consultation = 10 | | **Average direct medical costs per RA prescription per month in Indian Rupees 2017:**  997  **Average direct medical cost per patient per year in Indian Rupees (2017):** 11,965 | | **Average direct medical cost per patient per year adjusted in USD 2021:**  1008 | |
| **Kuijper et al. 2014 (6)** | Rotterdam, Netherlands  N=244^§§^  **Study duration:**  2004 – June 2009  **Funding source:**  Dutch Arthritis Foundation | **Objective**:  Comparison of disease burden between RA patients and arthralgia in an early arthritis cohort.  **Study design:**  Inception cohort study.  **Study setting:** Patients recruited at first consultation with general practitioners or Rheumatology outpatient of five hospitals. | **N**: 244 (F: 68%)  **Symptom duration at study inclusion*:**  103 (7-373) days  **Age**:  54 (SD 13.7)  **RF:** NA | **Outcome:**  Health care utilisation (number of visits)   1. GP 2. Specialist 3. Physiotherapist 4. Alternative     **Time points assessment:**  Baseline, six months, 12 months.  **Questionnaire recall time:** six months**.** | **Source of resources and source of costs:**  Resources: Self-reported questionnaire.  Costs: not done  **Perspective**: Healthcare | **Health care utilisation (HCU)**  **Baseline** (number of visits)   1. GP 2.8 visits 2. Specialist visits 1.4 3. Physiotherapist visits/5 = 0.5 4. Alternative visits = 0.1   All visits: 4.7units   1. **6 months** 2. GP 0.5 visits 3. Specialist visits 2.6 4. Physiotherapist visits/5 = 0.6 5. Alternative visits = 0.1   All visits= 3.9units   1. **12 months** 2. GP 0.4 visits 3. Specialist visits 1.6 4. *Physiotherapist visits/5 = 0.5 5. Alternative visits = 0.1   All visits= 2.6units | | **Combined Health Care Utilisation (HCU) units for the first 12 months post DMARD initiation:**  6.5 visits/pt/year | | Monetary value not reported | |
| **Puolakka et al. 2009 ^§§§^ (7)** | Finland,  N=159.  **Study duration**:  1993-2000  **Funding source:**  Paulo Foundation and Medicare Foundation | **Objective:**  Impact of HAQ on productivity loss in early RA patients.  **Study design:**  Data collection at 5-year follow-up in an extension of a randomised controlled trial.  **Study setting:**  18 recruitment centres for FIN-RACo Trial. | **HAQ GROUP 1**  Disease duration  at inclusion**:**  11(9) months  **N=**13 (F: 31%)  **Age:** 45 (SD 9)  **RF:** 77% | **Outcome:**   1. Work disability days 2. Indirect costs:   Loss of productivity per year by:   1. Human capital approach 2. Friction cost approach | **Data source for work disability**: Official register for sick leave and work disability pensions.  **Sources for costs:** Loss of productivity costs were converted to 2006 price by general index of wages and salaries ([www.stat.fi](http://www.stat.fi)).  **Human capital approach (HCA)** = number of hours lost in paid work (due to sick leave or work disability).  **Friction cost approach (FCA)** = production lost related to the 'friction period' i.e. the period after which the sick worker is usually replaced (in this study 230 days).  **Calculation** for HCA and FCA described in legend ѱѱ.  **Perspective:** Partial societal | **Values given as mean (95% CI)**  **HAQ GROUP 1:**  Work disability (days per year)  34 (5-145)  Loss of productivity per year (HCA),euros:  440 (137-896)  Loss of productivity per year (FCA), euros:  353 (118-712) | | | | **Loss of productivity per year in USD 2021, mean**  HCA: 736  FCA: 590 | |
|  |  |  | **HAQ GROUP 2**  Disease duration  at inclusion:  8 (5) months  **N=**65(F: 62%)  **Age:** 45 (SD 9)  **RF:** 71% |  |  | **HAQ GROUP 2:**  Work disability (days per year)  33 (19-57)  Loss of productivity per year (HCA) euros:  2704 (1457- 4606)  Loss of productivity per year (FCA), euros:  1360 (963-1870) | | | | **Loss of productivity per year in USD 2021, mean**  HCA: 4523  FCA: 2275 | |
|  |  |  | **HAQ GROUP 3**  Disease duration  at inclusion:  8 (5) months  **N=**65 (F: 68%)  **Age:** 47 (SD 4)  **RF:** 74% |  |  | **HAQ GROUP 3:**  Work disability (days per year)  146 (112-185)  Loss of productivity per year (HCA), euros:  12072 (8788-15758)  Loss of productivity per year (FCA), euros:  2452 (1902-3153) | | | | **Loss of productivity per year in USD 2021, mean**  HCA: 20191  FCA: 4101 | |
|  |  |  | **HAQ GROUP 4**  Disease duration  at inclusion:  10 (7) months  **N=**16 (F: 69%)  **Age:** 50 (SD 9)  **RF:** 56% |  |  | **HAQ GROUP 4:**  Work disability (days per year)  272 (194-328)  Loss of productivity per year (HCA), euros  23985 (16448-33141)  Loss of productivity per year (FCA), euros  3662 (2518-5237) | | | | **Loss of productivity per year in USD 2021, mean**  HCA: 40116  FCA: 6125 | |
| **Verstappen et al. 2004 10** | Utrecht, Netherlands  N: 509  **Study setting:**  Seven rheumatology OPA clinic in the Utrecht region^ѱ^  **Study duration:**  Cohort 1:  Recruited between Jan 1990 – April 1994  Cohort 2: Commenced 1998  **Funding source:**  Dutch Arthritis Association | **Objective:**  Estimation of annual direct costs and their predictors in patients with four disease duration groups.  **Study design:**  Cost-of-illness study within open-label extension of two randomised clinical trials.  Patients in RCT 1 were randomly assigned to 1 of 4 treatment regimes^§^.  Patients in RCT 2 were allocated to either intensive or conservative methotrexate treatment.[Questionnaires were sent out in Oct 1999 and April 2000.] | N=96 from group with disease duration follow-up: 0 to ≤2 years  **(F: 73%)**  **Disease duration at**  **inclusion** : 0.9 (0.6) year  Age: 54 (15)  **RF:** NA | **Outcome:**  Direct medical costs   1. Consultations with health care workers 2. Admissions to health care facilities (hospital including surgical procedures, rehab centre, nursing home) 3. Medication 4. Laboratory tests 5. Devices to perform daily activities and adaptations at home. 6. Alternative medicine 7. Other costs   **Measure method:** Self-reported questionnaire with 3 months recall | **Source of resource use and source of costs:**  **1**. Admission to care facilities, consultations with health care workers and travel expenses:   - Dutch guidelines for pharmaco-economic COI studies.   2. Surgical procedures and lab tests:   - Dutch college rates.  1. Costs of medication:  - Official drug price.   4. Costs for adaptations, devices, alternative medicines and other costs,   - Patient self-reported costs.   **Perspective**: Healthcare and patient | **Currency:** Euros; publication year 2004.  **Unit:** Mean (median) (range)   1. Consultation with healthcare workers:   1448 (1433) (0-8090)   1. Admission to care facilities:   1391 (7283)(0-57930)   1. Total hospital costs 376 (1348) 2. Hospitalisation 376 (1348) 3. Surgery 0 4. Rehabilitation centre 1015 (6383) 5. RA related medication 478 (406)(0-2895) 6. Devices and adaptations   963 (2247)(0-15571)   1. Laboratory tests   296 (131)(75-975)   1. Alternative therapies   103 (338)(0-6080)   1. Total extra costs   554 (1094) (0-6080)   1. Additional energy costs 42 (111) 2. Diet 20 (87) 3. Over the counter medication 13 (49) 4. Homeopathy 72 (276) 5. Health food 12 (101) 6. Payment to friends for help 15 (61) 7. Maintenance in and around the house 153 (570) 8. Clothing 51 (301) 9. Telephone 26 (102) 10. Parking permit 2(130) 11. Transportation 126 (345) 12. Other costs 0 | | **Direct costs per patient per year**  **Unit:** Mean (median) (range)  5235 (2923) (570-74080) | | **Mean of total direct costs per patient per year in USD 2021:**  14,613  **Median of total direct costs per patient per year in USD 2021:** 8159 | |
| **Merkesdal et al. 2001 11** | Germany. N=133.  **Study duration**: 3 Years  **Funding source**:  German Ministry of research and Technology. | **Objective:** To assess  1) the extent of indirect costs,  2) changes in cost components, and  3) correlations between changes in cost and social, clinical, and occupational variables within the first 3 years of RA.  **Design:** Longitudinal prospective observational study.  **Study setting: Four centres**: Medical School of Hannover, Ev. Fachkrankenhaus Ratingen, Klinik Niedersachsen Bad Nenndorf and Klinikum Sudstadt Rostock | **N**: 133 (F: 63)  **Disease duration at inclusion, mean (SEM):** 7 months (0.3).  **Age, mean (SEM):** 47 (0.8)  **RF:** 56%. | **Outcome:**  1. Indirect costs. Loss of productivity due to  i. sick leave  ii. work disability   1. other work loss   **Time-points of clinical assessments:**   1. Baseline, 2. 12 months, 3. 24 months.   **Measure method:**   1. Demographic, occupational, social variables, sickness-related workdays lost, income: Standardised interview 2. **Recall time-point:** 12 months. | **Data source for work disability**: Standardised interview.  **Valuation of loss of productivity:** Guidelines for economic evaluation using population data.  **Study perspective**: Partial societal | **Currency:** US dollars for the period 1994-1996.  Unit : Mean (SEM)  **Sick leave**  Time 0 – time 2: 10530 (990).  Time 2 – time 3: 2520 (580).  Time 0 – time 3: 7640 (740).  **Work disability**  Time 0 – time 2: 1210 (360).  Time 2 – time 3: 4570 (960).  Time 0 – time 3: 2520 (550).  **Other work loss**  Time 0 – time 2: 840 (370).  Time 2 – time 3: 2800 (780).  Time 0 – time 3: 1590 (480).  **Definition of time-point:**  Time 0 = Joint swelling onset.  Time 2 = 12 months from study enrolment.  Time 3 = 24 months from study enrolment. | | **Currency:** US dollars for the period 1994-1996.  **Total sick leave, work disability & other work loss**  **Unit:** Mean (SEM)  Time 0 – time 2:  12,580 (1030).  Time 2 – time 3:  9890 (1210).  Time 0 – time 3:  11,750 (1120). | | **Cost per person per year in USD 2021 after adjusting for purchasing power parity and Consumer Price Index 2021**  **Total sick leave, work disability & other work loss**  **Unit**: Mean  Time 0–time 2: 20,180  Time 2–time 3: 15, 865  Time 0–time 3: 18, 848 | |
| **Newhall-Perry et al. 2000 13** | The USA.  N=150.  **Recruitment period:** March 1993 to June 1996.  **Funding source:**  National Institute of Health Grant, Arthritis Foundation and Charlotte Mundt Trust | **Objective:** To examine direct and indirect costs of RA during the first year of disease.  **Design:** Longitudinal observational study.  **Study setting:** Patient recruited at 26 Rheumatology centres in western US and Mexico. (3 practices are University medical centres, 23 community practices. | **N:** 150 (F: 80%)  **Disease duration at inclusion:** 5.9 months (SD 2.9 months)  **Age:** 51 (SD 13)  **RF:** Not stated (Inclusion criteria states RF positivity) | **Outcome:**   1. Direct costs 2. Indirect costs   **Time-point of clinical assessments:** Baseline  **Measure method:**  Self-administered questionnaires  **Questionnaires recall time-point:** 6 months. | **Direct costs:**   1. **Medication costs:** average wholesale prices 1994 Red Book. 2. **Physician services, medical and surgical procedures:** Physicians’ Current Procedural Terminology ‘94 handbook. 3. **Podiatric care and procedures, radiological tests**: Medicare 1994 Fee Schedule. 4. **Laboratory tests:** Medicare reimbursement values. 5. **Hospitalisation costs:** billing information from patient. 6. **Costs of assist devices:** medical equipment wholesale catalogue. 7. **Costs of in-home assistance:** estimated wage of $55/hour. 8. **Non-traditional treatments:** patient self-reported.   **Indirect costs:**  **Employed individuals:**  1. Patient-reported annual income at the time of study enrolment.   1. Number of days of productive work lost due to arthritis.   **Unemployed individuals:**   1. Estimated based using median age, sex, educational attainment-specific employment incomes derived from the Current Population Reports. 2. Number of days of usual activity reported lost because of arthritis.   **Study perspective:**  Healthcare (direct costs)  Partial societal (indirect costs) | **Disease duration**  **<6 months** (n=87)  **Currency:** US dollars 1994  **Unit:** Mean (SD).  **Direct costs/month**240 (285)   1. Medication costs: 62 (101) 2. OTC 2(6) 3. Prescription 60 (101).   **2.** Healthcare visits: 65 (69)   1. Physician visits 54(48). 2. Other healthcare providers 11(43). 3. Radiographs 65 (196). 4. Laboratory tests: 27 (26). 5. Hospitalisations 0(0). 6. Assistive devices 3(6). 7. Non-traditional treatments 1(3). 8. In-home assistance 9 (47) 9. Outpatient procedures 8(49)   **Indirect cost per month** 348 (567)  **Disease duration ≥6 months**  Unit: Mean (SD).  **Direct costs/month** 144 (149)  1. Medications costs: 43(36)  i. OTC 1 (3)  ii. Prescription 42 (36)  2. Healthcare visits 37 (28)  i. Physician visits  30 (22)  ii. Other healthcare providers 6 (18)  3. Radiographs 26 (30)  4. Laboratory tests 13 (12)   1. Hospitalisations   16 (97)  6. Assistive devices 3 (11).   1. Non-traditional   treatments 2 (9)  8. In-home assistance 3 (16)  9. Outpatient procedures 1 (5)  **Indirect cost per** month 188 (506) | **Results in local currency and year of assessment**  Unit: Mean (SD)  **Total RA cost** (direct & indirect cost/month) ) in patients with disease duration < 6 months: 586 (686)  **Total RA cost** (direct & indirect cost/month) in patients with disease duration ≥ 6 months: 332 (585) | | **Cost per person per year in USD 2021 after adjusting for purchasing power parity and Consumer Price Index 2021**  **Total costs (direct and indirect costs) of RA per year per patient for overall cohort**, **mean:** 10,372  Direct costs per year per patient for overall cohort , mean: 4,322  Indirect costs of per year per patient for overall cohort , mean: 6,072  *Cost by disease duration groups:*  Indirect costs < 6 months, mean: 7,520  Indirect costs ≥ 6 months, mean: 4,063  Direct costs  < 6 months, mean: 5,186  Direct costs  ≥ 6 months, mean: 3,112  **Total costs (Direct and indirect) ,**  < 6 months, mean: 12,663  **Total cost (Direct and indirect)** ≥ 6 months, mean: 7174 | |  |
| **Van Jaarsveld et al. 1998 (8)** | Utrecht, Netherlands  N=363  **Study setting:**  Six rheumatology centres in Utrecht region.  **Study duration**:  1990 – April 1996  **Funding source:**  Dutch league against Rheumatism grant | **Objective:**  Estimation of:  1. Annual direct RA related costs in the first 6 years.  2. Socio-demographic and clinical predictors of these costs.  **Study design:**  Cross-sectional data collection of direct costs for all patients recruited in randomised clinical trial.  [Questionnaire sent in April 1996. First patient in trial was enrolled 1990. Results represented as the total group independent of the treatment arm. ] | N= 63 from patient with symptom duration at 1 year follow-up.  **F:** 64%  **Disease duration at inclusion:** 0-1 year  **Age:** 57 (19-84)*  **RF**: 66% | **Outcome:**  1. **Direct medical cost:**   1. Healthcare workers cost 2. Days in care facilities 3. Medication 4. Medication side effects monitoring 5. Alternative medicine   2. **Direct non-medical costs**  i. Devices and adaptations at home  ii. Other costs: travel expenses, medication not provided by national health service, additional costs of energy, telephone and clothing, payments to friends for care, payment for help around the house, and other costs specified by the patients.  **Measure method:**   1. Self-reported questionnaire 2. Hospital database 3. Clinical trials database   **Questionnaire time-point:** Self-reported questionnaire with 3 months recall | **Source of resource use and source of costs:**  1. Contact with healthcare providers:   - Self-reported questionnaires.   2. Days in care facilities:   - National costs of Dutch hospitals and nursing homes.   3. Medication costs:   - National pharmacy-therapeutical catalogue 1996.   4.Alternative medicine:   - Use and costs self-reported by patient.   5. Adaptations and devices at home using price list:   - Dr Butler catalogue: simple solution for daily discomfort; 1996. - Complete Ableware catalogue: aids for daily living & clinical modalities.1996. - Nottingham rehab catalogue. 1996   6. Other direct costs:   - Self-reported costs.   **Perspective:** Healthcare and patient | **Currency:** Dutch Florins; Sept 1997.  **Direct medical costs for disease duration 0-1year**  Mean (SD) Median per patient per year  Total direct cost  14455 (20411) 7370  Subtotal direct medical cost†  9882 (1898) 4444   1. Consultations with Health care worker   3355 (3112) 2340   1. Days in care facilities   4620 (15521) 0   1. Medication   1340 (682) 1170   1. Monitoring for side-effects   484 (311) 416   1. Alternative medicine   83 (299) 0  Subtotal direct non-medical cost  4573 (8934) 2268  Adaptations and devices  2814 (6797) 150   1. Other costs   1759 (3101) 600 | | **Direct medical cost for disease duration 0-1year**  **Mean (SD) Median** per patient per year in Dutch florins (Dfl)  **Total direct costs**  14455 (20411) 7370  Subtotal direct medical cost†  9882 (1898) 4444  Subtotal direct non-medical cost  4573 (8934) 2268 | | **Cost per person per year in USD 2021 after adjusting for purchasing power parity and Consumer Price Index 2021**  **Mean (Median)** per patient per year in USD 2021 (for at the end of year 1 of follow-up.  **Total direct costs**  24,094 (12285)  **Subtotal direct medical cost†**  16472 (7407)  **Subtotal direct non-medical cost** 7623 (3780) | |

^ѱ^Collaborating in the Utrecht RA cohort study group; ^§^Pyramid, IM gold, methotrexate or hydroxychloroquine; ^§§^ n = 330 arthralgia patients recruited; *median (range); §§§Outcome data were split into four groups based on HAQ: Group 1 (HAQ 0 at baseline and 6 m), Group 2 (HAQ>0 at baseline, 0 at 6m), Group 3 (HAQ≥0 at baseline, >0 but <1.0 at 6m), Group 4 (HAQ≥0 at baseline, ≥1.0 at 6m); †Subtotal of medical cost includes costs due to contacts with health care workers, days spent in care facilities, medication, monitoring for side effects and alternative medicine. Subtotal of non-medical direct cost includes costs of adaptations in the home, devices and other costs. Ѱ Ѱ HCA= Mean productivity per day over a five-year follow-up was calculated for each patient and multiplied by the cumulative number of their days off work to yield the patients' loss of productivity by the HCA. FCA= estimation of loss of productivity with the assumption that someone replaces the disabled worker after the friction period, and the initial production level is restored, that production losses are confined to the friction period**.** RA-related work disability days were obtained from the official register, divided by the duration (in years) of follow-up during which the patient had not retired due to other diseases or because of age. All final cost column states the cost per person per year in USD 2021 after adjusting for purchasing power parity and Consumer Price Index 2021.

# Supplementary Data S3. Quality assessment

Quality assessment was based on a modified checklist described by Drummond and Jefferson (3) and The Strengthening the Reporting of Observational Studies in Epidemiology (STROBE) (2) (supplementary table S1). All studies justified the rationale and scientific background for their investigation. Puolakka (7), Van Jaarsveld (8) and Merkesdal's (9) papers scored highly on analysis and interpretation of results. The majority of the studies commented on the external validity of their results and provided a balanced discussion incorporating key results, limitations and in the context of other relevant studies. Only three studies clearly stated and justified the viewpoint of the analysis (10-12)

# Supplementary Table S3. Quality assessment based on modified checklist described by Drummond and Jefferson (3) and STROBE (2) checklists.

| **Modified checklist described by**  **Drummond and Jefferson**  **items (3)** | **Luurssen-Masurel 2021 (14)** | **Verhoeven 2021 (15)** | **Syngle et al. 2017 (16)** | **Kuijper et al. 2014(6)** | **Puolakka et al. 2009(7)** | **Verstappen et al. 2004 (10)** | **Merkesdal et al. 2001 (11)** | **Newhall- Perry et al. 2000 (13)** | **Van Jaarsveld et al. 1998 (8)** |
| --- | --- | --- | --- | --- | --- | --- | --- | --- | --- |
| **Study design** |  |  |  |  |  |  |  |  |  |
| The research question is stated | Yes | Yes | Yes | Yes | Yes | Yes | Yes | Yes | Yes |
| The economic importance of the research question is stated | Yes | Yes | Yes | Yes | Yes | Yes | Yes | Yes | Yes |
| The viewpoint(s) of the analysis are clearly stated and justified | Yes | Yes | No | No | Partly | Yes | No | Partly | Partly |
| **Data collection** |  |  |  |  |  |  |  |  |  |
| The primary outcome measure(s) for the economic evaluation are clearly stated | Yes | Yes | Partly | Yes | Yes | Yes | Yes | Yes | Yes |
| Productivity changes (if included) are reported separately | Yes | Yes | NA | NA | Yes | NA | Yes | NA | NA |
| The relevance of productivity changes to the study question is discussed | Partly | No | NA | NA | Yes | NA | Partly | NA | NA |
| Quantities of resources are reported separately from their unit cost | Yes | No | Partly | NA | Yes | Yes | Yes | No | Yes |
| Methods for the estimation of quantities and unit costs are described | Yes | Yes | Yes | NA | Yes | Yes | Yes | Yes | Yes |
| Currency and price data are recorded | Yes | Yes | Yes | NA | Partly | Yes | Yes | Yes | Yes |
| Details of price adjustments for inflation or currency conversion are given | No | No | No | NA | No | Yes | Yes | Yes | Yes |
| **Analysis and interpretation of results** |  |  |  |  |  |  |  |  |  |
| Time horizon of costs and benefits is stated | Yes | Yes | Yes | Yes | Yes | Yes | Yes | Yes | Yes |
| The approach to sensitivity analysis is given | Yes | Yes | No | NA | Yes | Yes | Yes | No | Yes |
| The choice of variables for sensitivity analysis is justified | No | Yes | No | NA | Yes | Partly | Yes | No | Yes |
| The ranges over which the variables are varied are stated | Partly | Yes | No | NA | Yes | Yes | Yes | Yes | Yes |
| The details of statistical tests and confidence intervals are given for stochastic data | Partly | Partly | Partly | Yes | Yes | Yes | Yes | Yes | Yes |
| The major outcomes are presented in a disaggregated as well as aggregated form | Yes | Yes | Yes | Yes | Yes | Yes | Yes | Yes | Yes |
| The answer to the study question is given | Yes | Yes | Yes | Yes | Yes | Yes | Yes | Yes | Yes |
| Conclusions follow from the data reported | Yes | Yes | Yes | Yes | Yes | Yes | Yes | Yes | Yes |
| Conclusions are accompanied by the appropriate caveats | Yes | Yes | Yes | Yes | Yes | Yes | Yes | Yes | Yes |

| **STROBE checklist (2)** | **Luurssen-Masurel**  **2021 (14)** | **Verhoeven 2021 (15)** | **Syngle et al. 2017 (16)** | **Kuijper et al. 2014(6)** | **Puolakka et al. 2009(7)** | **Verstappen et al. 2004 (10)** | **Merkesdal et al. 2001 (11)** | **Newhall- Perry et al. 2000 (13)** | **Van Jaarsveld et al. 1998 (8)** |
| --- | --- | --- | --- | --- | --- | --- | --- | --- | --- |
| **Title and abstract** |  |  |  |  |  |  |  |  |  |
| (*a*) Indicate the study’s design with a commonly used term in the title or the abstract | Yes | Yes | Yes | Partly | Yes | Partly | Yes | Yes | Yes |
| (*b*) Provide in the abstract an informative and balanced summary of what was done and what was found | Yes | Yes | Yes | Yes | Yes | Yes | Yes | Yes | Yes |
| **Introduction** |  |  |  |  |  |  |  |  |  |
| Explain the scientific background and rationale for the investigation being reported | Yes | Yes | Yes | Yes | Yes | Yes | Yes | Yes | Yes |
| State specific objectives, including any prespecified hypotheses | Partly | Partly | Partly | Yes | Yes | Yes | Yes | Yes | Yes |
| **Methods** |  |  |  |  |  |  |  |  |  |
| Present key elements of study design early in the paper | Yes | Yes | Yes | Yes | Yes | Yes | Yes | Yes | Yes |
| Describe the setting, locations, and relevant dates, including periods of recruitment, exposure, follow-up, and data collection | Partly | Yes | Yes | Partly | Yes | Yes | Yes | Yes | Yes |
| (*a*) *Cohort study*—Give the eligibility criteria, and the sources and methods of selection of participants. Describe methods of follow-up  *Case-control study*—Give the eligibility criteria, and the sources and methods of case ascertainment and control selection. Give the rationale for the choice of cases and controls  *Cross-sectional study*—Give the eligibility criteria, and the sources and methods of selection of participants | NA | NA | Partly | Partly | Yes | Yes | Yes | Yes | Partly |
| Clearly define all outcomes, exposures, predictors, potential confounders, and effect modifiers. Give diagnostic criteria, if applicable | NA | NA | Partly | Yes | Yes | Yes | Partly | Partly | Partly |
| For each variable of interest, give sources of data and details of methods of assessment (measurement). Describe comparability of assessment methods if there is more than one group | Yes | Yes | Yes | Yes | Yes | Yes | Yes | Yes | Yes |
| Describe any efforts to address potential sources of bias | No | No | No | No | Yes | Yes | No | No | Partly |
| Explain how the study size was arrived at | No | No | No | No | No | No | No | No | No |
| **Quantitative** **variables**  Explain how quantitative variables were handled in the analyses. If applicable, describe which groupings were chosen and why | Yes | Yes | Partly | Yes | Yes | Yes | Yes | Yes | Yes |
| **Statistical** **methods**  (*a*) Describe all statistical methods, including those used to control for confounding | Yes | Yes | Partly | Yes | Yes | Yes | Yes | Yes | Yes |
| (*b*) Describe any methods used to examine subgroups and interactions | NA | NA | No | Yes | Yes | Yes | Yes | No | Yes |
| (*c*) Explain how missing data were addressed | Yes | Yes | No | Yes | No | Yes | No | No | No |
| (*d*) *Cohort study*—If applicable, explain how loss to follow-up was addressed  *Case-control study*—If applicable, explain how matching of cases and controls was addressed  *Cross-sectional study*—If applicable, describe analytical methods taking account of sampling strategy | NA | NA | No | No | Yes | NA | No | No | NA |
| (*e*) Describe any sensitivity analyses | Yes | Yes | No | Partly | Yes | Yes | Yes | No | Yes |
| **Results** |  |  |  |  |  |  |  |  |  |
| Participants  (a) Report numbers of individuals at each stage of study—e.g., numbers potentially eligible, examined for eligibility, confirmed eligible, included in the study, completing follow-up, and analysed | No | No | No | No | Partly | No | Yes | No | No |
| (b) Give reasons for non-participation at each stage | No | No | No | No | Yes | Yes | No | No | No |
| (c) Consider use of a flow diagram | No | No | No | No | No | No | No | No | No |
| **Descriptive data**  (a) Give characteristics of study participants (e.g, demographic, clinical, social) and information on exposures and potential confounders | Yes | Yes | Yes | Yes | Yes | Yes | Yes | Yes | Yes |
| (b) Indicate number of participants with missing data for each variable of interest | No | No | No | No | No | Partly | No | No | Yes |
| (c) *Cohort study*—Summarise follow-up time (eg, average and total amount) | NA | NA | Yes | Yes | Yes | Partly | Yes | No | No |
| ***Outcome data***  *Cohort study*—Report numbers of outcome events or summary measures over time  *Case-control study—*Report numbers in each exposure category, or summary measures of exposure  *Cross-sectional study—*Report numbers of outcome events or summary measures | NA | NA | Yes | Yes | Yes | NA | Yes | Yes | NA |
| **Main results**  (*a*) Give unadjusted estimates and, if applicable, confounder-adjusted estimates and their precision (e.g., 95% confidence interval). Make clear which confounders were adjusted for and why they were included | Yes | Yes | Partly | Yes | Partly | Yes | Yes | Yes | Yes |
| (*b*) Report category boundaries when continuous variables were categorized | NA | NA | No | Partly | Yes | Yes | No | Yes | No |
| (*c*) If relevant, consider translating estimates of relative risk into absolute risk for a meaningful time period | NA | NA | NA | NA | Yes | Partly | NA | NA | NA |
| **Other analyses**  Report other analyses done—e.g., analyses of subgroups and interactions, and sensitivity analyses | Yes | Yes | No | No | No | Yes | Partly | Yes | Yes |
| **Discussion** |  |  |  |  |  |  |  |  |  |
| Summarise key results with reference to study objectives | Yes | Yes | Partly | Yes | Yes | Yes | Yes | Yes | Yes |
| Discuss limitations of the study, taking into account sources of potential bias or imprecision. Discuss both direction and magnitude of any potential bias | Yes | Yes | Partly | Yes | Yes | Yes | Yes | Yes | Yes |
| Give a cautious overall interpretation of results considering objectives, limitations, multiplicity of analyses, results from similar studies, and other relevant evidence | Yes | Yes | Partly | Yes | Yes | Yes | Yes | Yes | Yes |
| Discuss the generalisability (external validity) of the study results | Yes | Partly | Yes | Yes | Yes | Yes | No | Yes | Yes |
| **Other information** |  |  |  |  |  |  |  |  |  |
| Give the source of funding and the role of the funders for the present study and, if applicable, for the original study on which the present article is based | Yes | Partly | Yes | Yes | Yes | Yes | Yes | Yes | Yes |

Yes: fulfill quality assessment criteria; No: did not fulfil quality assessment criteria; partly fulfill quality assessment criteria.
